# Elasticity of a Grafted Rod-like Filament with Fluctuating Bending Stiffness

**DOI:** 10.3390/polym15102307

**Published:** 2023-05-15

**Authors:** Mohammadhosein Razbin, Panayotis Benetatos

**Affiliations:** 1 Department of Energy Engineering and Physics, Amirkabir University of Technology, Tehran 14588, Iran; 2 Department of Physics, Kyungpook National University, 80 Daehakro, Bukgu, Daegu 41566, Republic of Korea

**Keywords:** wormlike chain, elasticity, statistical ensemble inequivalence, negative compressibility

## Abstract

Quite often polymers exhibit different elastic behavior depending on the statistical ensemble (Gibbs vs. Helmholtz). This is an effect of strong fluctuations. In particular, two-state polymers, which locally or globally fluctuate between two classes of microstates, can exhibit strong ensemble inequivalence with negative elastic moduli (extensibility or compressibility) in the Helmholtz ensemble. Two-state polymers consisting of flexible beads and springs have been studied extensively. Recently, similar behavior was predicted in a strongly stretched wormlike chain consisting of a sequence of reversible blocks, fluctuating between two values of the bending stiffness (the so called reversible wormlike chain, rWLC). In this article, we theoretically analyse the elasticity of a grafted rod-like semiflexible filament which fluctuates between two states of bending stiffness. We consider the response to a point force at the fluctuating tip in both the Gibbs and the Helmholtz ensemble. We also calculate the entropic force exerted by the filament on a confining wall. This is done in the Helmholtz ensemble and, under certain conditions, it yields negative compressibility. We consider a two-state homopolymer and a two-block copolymer with two-state blocks. Possible physical realizations of such a system would be grafted DNA or carbon nanorods undergoing hybridization, or grafted F-actin bundles undergoing collective reversible unbinding.

## 1. Introduction

The elasticity of semiflexible polymers has been the subject of intense research in recent years, because of their relevance to cell biology and nanotechnology, but also because of the inherent theoretical challenges that they pose. At the single molecule level, one of the peculiarities of semiflexible polymer elasticity is its dependence on the statistical ensemble (Gibbs or fixed force vs. Helmholtz or fixed extension).

The equivalence of statistical ensembles (microcanonical vs. canonical, Gibbs vs. Helmholtz, and so on) is in fact a consequence of the central limit theorem of probability theory and holds in the thermodynamic limit of systems with short-range interactions [1,2]. In the thermodynamic limit of such systems, the ratio of the standard deviation of fluctuating quantities over the corresponding average value vanishes, and fluctuations become negligible, thus yielding statistical ensemble equivalence. There are notable exemptions to this equivalence. The so-called self-gravitating systems are one example, where the ensemble inequivalence is the result of the long-range nature of the gravitational interaction. The behavior of polymers is dominated by fluctuations and ensemble inequivalence occurs quite often [3,4,5]. Here, we focus on the Gibbs and the Helmholtz ensembles.

Flexible, Gaussian chains are known to exhibit ensemble equivalence, as long as the two ensembles correspond to precisely conjugate variables (as they appear in the expression for the work) [6,7,8,9]. The freely jointed chain exhibits ensemble inequivalence if it has a finite number of segments and it exhibits ensemble equivalence in the thermodynamic limit [10]. The wormlike chain (WLC) is a widely used minimal model of semiflexible polymers. It is known to exhibit ensemble inequivalence [11,12], except for two extreme cases, namely, the weakly bending limit of a very stiff WLC with very large persistence length compared to the contour length, and the weakly bending limit of a strongly stretched WLC. The freely jointed chain can be viewed as an extreme case of a semiflexible polymer with infinite bending stiffness along the rods and zero bending stiffness at the hinges.

The polymers that we have mentioned so far are homopolymers, far away from any boundaries. It is known that ensemble inequivalence may also be the result of confinement (even in the case of Gaussian chains) [13,14,15,16,17,18,19]. In addition, another important class of polymers exhibits ensemble inequivalence, and the system analysed in the present article belongs to that class. These are two-state copolymers, consisting of a concatenated sequence of units which fluctuate between two states. There has been a significant body of literature analyzing flexible chains of bistable units [20,21,22,23,24,25,26,27,28]. Usually, the units are treated as harmonic springs which can have two different values of rest length and/or spring constant.

Recently, the study of the tensile elasticity of chains of bistable units was extended beyond the flexible case with the analysis of the so called reversible wormlike chain (rWLC) [29] in the strong stretching regime. The rWLC is a WLC consisting of a concatenated sequence of blocks of equal contour length that can be in one of two states, differing in the value of the bending stiffness. The rWLC was analyzed in the strong stretching approximation. It was proven that, in the thermodynamic limit, it exhibits ensemble equivalence, even though the force-extension relation differs significantly from that of the homopolymer (uniform) WLC case. A strongly stretched rWLC with a finite number of reversible blocks has a monotonic force-extension relation in the Gibbs ensemble (as required by the second law of thermodynamics), but, under certain conditions, it can have a spiky (non-monotonic) force-extension relation in the Helmholtz ensemble. In the present article, we consider a rWLC in the other realization of the weakly bending approximation. Here, the rWLC is in the stiff (rodlike) limit, grafted to a fixed substrate. We consider the case of one reversible block and that of two reversible blocks. We consider the response to a point force at the free tip and also to confinement by a rigid wall. The former case (for one reversible block) is treated both in the Gibbs and in the Helmholtz ensemble. The confinement average force is calculated in the Helmholtz ensemble.

In general, the two states of bistable polymers are the result of internal conformational changes (e.g., the helix-coil transition in polypeptides or the nucleation of denaturation bubbles in dsDNA) or they are related to the reversible binding/unbinding of ligands from the environment. The model that we analyse in the present article may be relevant to a DNA or carbon nanotube which becomes reversibly hybridized by a DNA chain [30,31,32]. The hybrid state has higher bending stiffness than the original one. Another physical system which may be related to our model is the actomyosin complex in the sarcomere [33,34]. As myosin attaches and detaches from two or more actin filaments, it changes the bending stiffness of the actin bundle, which in turn can be viewed on a coarse-grained level as a bistable rod-like semiflexible filament.

Our article is organized as follows. In Section 1, we review the rod-like (stiff) limit of a grafted wormlike chain and we calculate the linear response to a point force exerted at the fluctuating tip. We also calculate the entropic force due to confinement by a planar wall. In Section 2, we address the same questions about the elastic response of a weakly bending grafted wormlike chain, which fluctuates between two possible values of its bending stiffness. In Section 3, we analyse the elasticity of a grafted wormlike chain at the stiff limit which consists of two concatenated blocks of different bending stiffness. In Section 4, we consider the case where the two-block grafted stiff chain has a two-valued fluctuating bending stiffness in each block. We summarize and conclude in Section 5.

## 2. Elasticity of a Grafted Weakly Bending Semiflexible Homopolymer with Fixed Bending Stiffness

The “Hamiltonian” (effective free energy functional) of a two-dimensional wormlike chain (WLC) with bending stiffness κ is

(1) $$H_{WLC}=\frac{\kappa }{2}\int_{0}^{L}{\left(\frac{dt(s)}{ds}\right)}^{2}ds$$

where κ is the bending stiffness and t (s) is the tangent vector to the filament contour at arc length, *s*. In two dimensions, the persistence length of the chain (defined as the correlation length of the tangent vector along the polymer contour), $l_{p}$, has the following relation with the bending stiffness:

(2) $$l_{p}=\frac{2\kappa }{k_{B}T}\;,$$

where $k_{B}$ is the Boltzmann constant and *T* is the temperature. In this article, for the sake of simplicity in the calculations, we analyze a filament in two dimensions. However, the three-dimensional case will differ only in numerical prefactors because, in the weakly bending approximation, the two transverse dimensions decouple [35].

**Figure 1:**
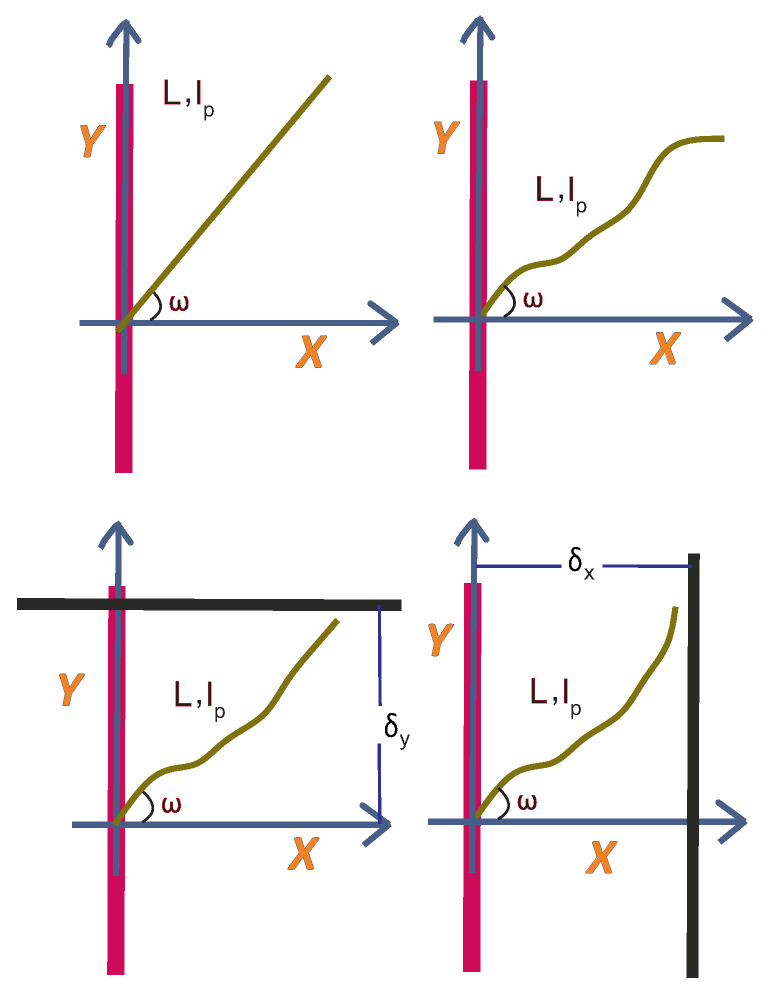
**Upper** panel: The zero temperature configuration of the grafted worm-like chain (WLC) in two dimensions (upper left panel). The filament is grafted to a stiff wall with a grafting angle ω and has a fixed contour length, *L*, and a fixed persistence length, $l_{p}$, at a finite temperature. The filament can fluctuate due to the thermal noise at equilibrium state (upper right panel). **Lower** panel: A stiff planar wall can confine the tip of the filament in the *x* direction (lower right panel) or in the *y* direction (lower left panel). The tip of the filament can exert force on the wall due to its reduction of configurational entropy.

In this section, we consider a grafted WLC (in two dimensions, as shown in Figure 1) in the stiff limit. Because of the large value of the bending rigidity, $L\ll l_{P}$, and the deflection away from the grafting direction is small, so that sin(θ−ω)≈θ−ω and cos(θ−ω)≈1. The positional-orientational propagator of the chain is denoted by $G_{L,l_{p}}\left(x,y,\theta |x_{0},y_{0},\omega \right)$. It is interpreted as the conditional probability density to find the endpoint of the chain at position (x,y) with orientation θ given that it is grafted at position $\left(x_{0},y_{0}\right)$ with orientation ω.

In the weakly bending regime, the propagator is calculated as a multivariate Gaussian expression [36],

(3) $$\begin{array}{c} G_{L,l_{p}}\left(x,y,\theta |x_{0},y_{0},\omega \right)=\frac{1}{N_{G}}\exp[-\frac{3l_{p}}{L^{3}}(\left(y-y_{0}\right)\cos\left(\omega \right)\\ -\left(x-x_{0}\right)\sin\left(\omega \right){)}^{2}-\frac{l_{p}}{L}{\left(\theta -\omega \right)}^{2}]\\ \times \exp[\frac{3l_{p}}{L^{2}}\left(\left(y-y_{0}\right)\cos\left(\omega \right)-\left(x-x_{0}\right)\sin\left(\omega \right)\right)\left(\theta -\omega \right)]\\ \times \delta [\left(x-x_{0}\right)\cos\left(\omega \right)+\left(y-y_{0}\right)\sin\left(\omega \right)-L]\;, \end{array}$$

where δ(x) is the Dirac δ-function and the factor $N_{G}$ is determined by the normalization condition,

(4) $$\begin{array}{c} \int \int \int dxdyd\theta \;G_{L,l_{p}}\left(x,y,\theta |x_{0},y_{0},\omega \right)=1\;. \end{array}$$

In the remainder of this article, we use the notation $\int \equiv \int_{-\infty }^{+\infty }$ for the sake of simplicity. Using Equation (3), we can easily calculate the probability density of the *x* component of the free endpoint position,

(5) $$\begin{array}{cc} P_{x}\left(x\right) & =\int \int dyd\theta G_{L,l_{p}}\left(x,y,\theta |0,0,\omega \right)\\ & =\sqrt{\frac{3l_{p}}{4\pi L^{3}\sin^{2}\left(\omega \right)}}\exp\left(-\frac{3l_{p}{\left(x-L\cos\left(\omega \right)\right)}^{2}}{4L^{3}\sin^{2}\left(\omega \right)}\right)\;. \end{array}$$

The probability density implies the following Helmholtz free energy:

(6) $$H_{x}\left(L,l_{p},\omega \right)=\frac{3k_{B}Tl_{p}{\left(x-L\cos\left(\omega \right)\right)}^{2}}{4L^{3}\sin^{2}\left(\omega \right)}\;.$$

From this equation, we readily obtain the force-extension relation in the fixed extension (Helmholtz) ensemble:

(7) $$\begin{array}{cc} \langle f_{x}\rangle & =\frac{\partial H_{x}\left(L,l_{p},\omega \right)}{\partial x}\\ & =\frac{3k_{B}Tl_{p}\left(x-L\cos\left(\omega \right)\right)}{2L^{3}\sin^{2}\left(\omega \right)}\;. \end{array}$$

Because in the stiff (weakly bending) limit the probability density is Gaussian, the chain exhibits ensemble equivalence. In the presence of a fixed force in the *x* direction, the free energy is obtained by the following Legendre transformation:

(8) $$\begin{array}{cc} G_{f}\left(L,l_{p},\omega \right) & =H_{x}\left(L,l_{p},\omega \right)-f_{x}x\\ & =-\frac{L^{3}\sin^{2}\left(\omega \right)}{3k_{B}Tl_{p}}f_{x}^{2}-f_{x}L\cos\left(\omega \right)\;. \end{array}$$

We use this Gibbs free energy and calculate the average of the *x* component of the endpoint position of the filament in the fixed force ensemble,

(9) $$\begin{array}{cc} \langle x\rangle & =-\frac{\partial G_{f}\left(L,l_{p},\omega \right)}{\partial f_{x}}\\ & =\frac{2L^{3}\sin^{2}\left(\omega \right)}{3k_{B}Tl_{p}}f_{x}+L\cos\left(\omega \right)\; \end{array}$$

Notice that it is identical to the force-extension relation in the fixed extension ensemble that we obtained above. In the Gibbs ensemble, we can obtain the same force-extension relation if we view $H_{x}\left(L,l_{p},\omega \right)-f_{x}x$ as the Hamiltonian of an effective harmonic spring subjected to a constant force,

(10) $$\begin{array}{cc} \langle x\rangle & =\frac{\int_{-\infty }^{+\infty }\left(x\right)\exp\left(\frac{-H_{x}\left(L,l_{p},\omega \right)}{k_{B}T}\right)dx}{\int_{-\infty }^{+\infty }\exp\left(\frac{-H_{x}\left(L,l_{p},\omega \right)}{k_{B}T}\right)dx}\\ & =\frac{2L^{3}\sin^{2}\left(\omega \right)}{3k_{B}Tl_{p}}f_{x}+L\cos\left(\omega \right)\; \end{array}$$

Now, we consider a stiff planar wall being placed in front of the tip of the filament. The position of the wall is fixed. The wall restricts the tip of the filament in the *x* direction which leads to a reduction in the configurational entropy of the grafted filament (see Figure 1). We are going to calculate the ensuing entropic force using the method originally introduced by Gholami et al. [37]. The fractional (relative) reduction in the number of configurations of the tip of the filament is given by the following expression:

(11) $$Z_{x}\left(\delta_{x}\right)=\frac{\int_{-\infty }^{\delta_{x}}\exp\left(\frac{-H_{x}\left(L,l_{p},\omega \right)}{k_{B}T}\right)dx}{\int_{-\infty }^{+\infty }\exp\left(\frac{-H_{x}\left(L,l_{p},\omega \right)}{k_{B}T}\right)dx}=\int_{-\infty }^{\delta_{x}}P_{x}\left(x\right)dx$$

The force on the wall due to the reduction in the number of configurations of the filament by the presence of the wall is obtained by the derivative of the logarithm of corresponding partition function,

(12) $$\langle f_{x}^{PW}\rangle =k_{B}T\frac{d}{d\delta_{x}}\ln\left(Z_{x}\left(\delta_{x}\right)\right)$$

The derivative in the above equation gives the average force exerted on the wall by the fluctuating tip of the filament in the *x* direction,

(13) $$\langle f_{x}^{PW}\rangle =-\sqrt{\frac{3K_{x}}{\pi }}\left(\frac{k_{B}T\exp\left(-\frac{3K_{x}{\left(L\cos\left(\omega \right)-\delta_{x}\right)}^{2}}{4}\right)}{\mathrm{erf}\left(\frac{\sqrt{3K_{x}}}{2}\left(L\cos\left(\omega \right)-\delta_{x}\right)\right)-1}\right)\;,$$

where

(14) $$K_{x}\equiv \frac{l_{p}}{L^{3}\sin^{2}\left(\omega \right)}$$

We now consider the elastic response in the *y* direction. The probability density of the *y* component of the free endpoint position is

(15) $$\begin{array}{cc} P_{y}\left(y\right) & =\int \int dxd\theta G_{L,l_{p}}\left(x,y,\theta |0,0,\omega \right)\\ & =\sqrt{\frac{3l_{p}}{4\pi L^{3}\cos^{2}\left(\omega \right)}}\exp\left(-\frac{3l_{p}{\left(y-L\sin\left(\omega \right)\right)}^{2}}{4L^{3}\cos^{2}\left(\omega \right)}\right)\;. \end{array}$$

This probability density implies the following expression for the Helmholtz free energy in the *y* direction:

(16) $$H_{y}\left(L,l_{p},\omega \right)=\frac{3k_{B}Tl_{p}{\left(y-L\sin\left(\omega \right)\right)}^{2}}{4L^{3}\cos^{2}\left(\omega \right)}\;.$$

The force-extension relation in the *y* direction in the Helmholtz ensemble is

(17) $$\begin{array}{cc} \langle f_{y}\rangle & =\frac{\partial H_{y}\left(L,l_{p},\omega \right)}{\partial y}\\ & =\frac{3k_{B}Tl_{p}\left(y-L\sin\left(\omega \right)\right)}{L^{3}\cos^{2}\left(\omega \right)}\;. \end{array}$$

In the presence of the fixed force in the *y* direction, $f_{y}$, the free energy can be treated as an effective Hamiltonian:

(18) $$\begin{array}{c} H_{y}^{\prime }\left(L,l_{p},\omega \right)=\frac{3k_{B}Tl_{p}{\left(y-L\sin\left(\omega \right)\right)}^{2}}{4L^{3}\cos^{2}\left(\omega \right)}-f_{y}y \end{array}$$

The average of the *y* component of the end point position is given by the Boltzmann weighted average in the fixed force (Gibbs) ensemble,

(19) $$\begin{array}{c} \langle y\rangle =\frac{\int_{-\infty }^{+\infty }\left(y\right)\exp\left(-\frac{H_{y}^{\prime }\left(L,l_{p},\omega \right)}{k_{B}T}\right)dx}{\int_{-\infty }^{+\infty }\exp\left(-\frac{H_{y}^{\prime }\left(L,l_{p},\omega \right)}{k_{B}T}\right)dx}\;. \end{array}$$

We obtain the force-extension relation in the *y* direction in the fixed force ensemble by calculating the integrals in the above equation,

(20) $$f_{y}=\frac{3}{2}\frac{k_{B}Tl_{p}\left(\langle y\rangle -L\sin\left(\omega \right)\right)}{L^{3}\cos^{2}\left(\omega \right)}\;.$$

Now, we consider the effect of putting a stiff planar wall in front of the tip of the filament in the *y* direction. The wall restricts the tip of the filament in the *y* direction. The fractional reduction in the number of the configurations of the tip of the filament in the presence of such a wall is given by the following expression:

(21) $$Z_{y}\left(\delta_{y}\right)=\frac{\int_{-\infty }^{\delta_{y}}\exp\left(-\frac{H_{y}\left(L,l_{p},\omega \right)}{k_{B}T}\right)dy}{\int_{-\infty }^{+\infty }\exp\left(\frac{-H_{y}\left(L,l_{p},\omega \right)}{k_{B}T}\right)dy}=\int_{-\infty }^{\delta_{y}}P_{y}\left(y\right)dy$$

The related force is given by

(22) $$\langle f_{y}^{PW}\rangle =k_{B}T\frac{d}{d\delta_{y}}\ln\left(Z_{y}\left(\delta_{y}\right)\right)$$

The derivative in the above equation gives the average force exerted on the wall by the fluctuating tip of the filament in the *y* direction,

(23) $$\langle f_{y}^{PW}\rangle =-\sqrt{\frac{3K_{y}}{\pi }}\left(\frac{k_{B}T\exp\left(-\frac{3K_{y}{\left(L\sin\left(\omega \right)-\delta_{y}\right)}^{2}}{4}\right)}{\mathrm{erf}\left(\frac{\sqrt{3K_{y}}}{2}\left(L\sin\left(\omega \right)-\delta_{y}\right)\right)-1}\right)\;,$$

where

(24) $$K_{y}\equiv \frac{l_{p}}{L^{3}cos^{2}\left(\omega \right)}\;.$$

We point out that, in the calculation of the confinement forces, we consider only collisions of the fluctuating tip with the confining wall and we neglect the possibility of collisions from the other points along the polymer contour. For stiff chains within the weakly bending approximation, this approximation seems reasonable.

## 3. Grafted Rod-like Semiflexible Filament with Fluctuating Persistence Length

**Figure 2:**
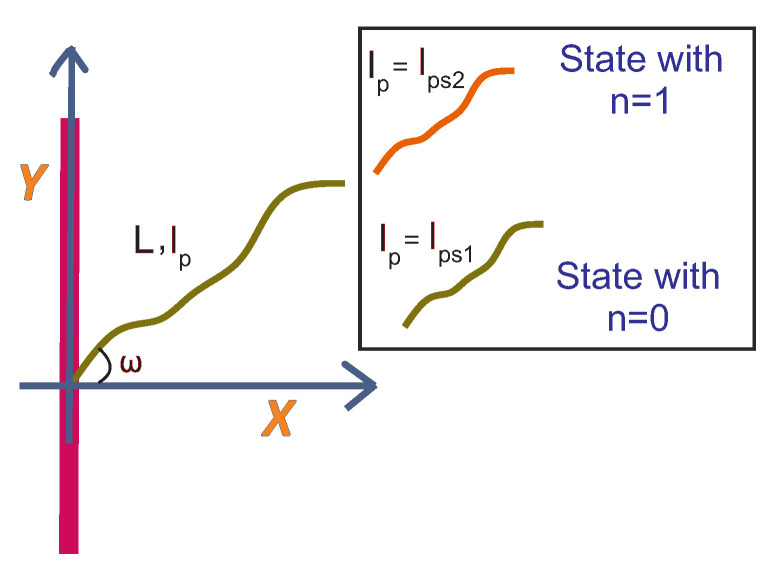
The grafted filament with a fluctuating persistence length at a finite temperature. The filament can be in two states associated with the two persistence lengths such as $l_{p}=l_{ps1}$ and $l_{p}=l_{ps2}$. The first state is labelled with n=0 and the second state is labelled with n=1. The transition from the first state to the second one requires a fixed energetic cost which is denoted by $\epsilon =k_{B}T\tilde{\epsilon }$.

We consider a wormlike filament with fluctuating persistence length. The fluctuation of the persistence length happens between two states in a stochastic thermal (equilibrium) process. The first state with persistence length, $l_{ps1}$ refers to the case with a value equal to zero for the Ising-like variable, n=0, while the second state refers to the case with n=1. The transition from the first state to the second one is accompanied by an energetic cost that is denoted by $\epsilon \equiv \tilde{\epsilon }k_{B}T$. A schematic illustration of this filament is shown in Figure 2. The Hamiltonian of such a filament in a state characterized by a certain conformation t(s) and a certain value of the Ising-like variable *n* is as follows:

(25) $$H_{WLC}^{\left(n\right)}\left\{t\left(s\right)\right\}=\frac{k_{B}T\left(l_{ps1}+n\left(l_{ps2}-l_{ps1}\right)\right)}{4}\int_{0}^{L}{\left(\frac{dt(s)}{ds}\right)}^{2}ds+n\epsilon \;.$$

The Helmholtz free energy associated with the endpoint position of the grafted filament in the *x* coordinate, for a fixed *n* state, is

(26) $$\begin{array}{cc} H_{x}^{\left(n\right)}\left(L,l_{ps1},l_{ps2},\omega \right) & =H_{x}\left(L,l_{ps1}+n\left(l_{ps2}-l_{ps1}\right),\omega \right)\\ & +n\epsilon \\ & =\left(\frac{3k_{B}T(l_{ps1}+n\left(l_{ps2}-l_{ps1}\right){\left(x-L\cos\left(\omega \right)\right)}^{2}}{4L^{3}\sin^{2}\left(\omega \right)}\right)\\ & +n\epsilon \end{array}$$

In the presence of the external force in the *x* direction, $f_{x}=k_{B}T\tilde{f_{x}}$, the free energy can be treated as an effective Hamiltonian of a spring under force:

(27) $$\begin{array}{cc} H_{x}^{\prime (n)}\left(L,l_{ps1},l_{ps2},\omega \right) & =H_{x}^{\prime }\left(L,l_{ps1}+n\left(l_{ps2}-l_{ps1}\right),\omega \right)\\ & +n\epsilon \\ & =\left(\frac{3k_{B}T(l_{ps1}+n\left(l_{ps2}-l_{ps1}\right){\left(x-L\cos\left(\omega \right)\right)}^{2}}{4L^{3}\sin^{2}\left(\omega \right)}\right)\\ & -f_{x}\left(x-L\cos\left(\omega \right)\right)+n\epsilon \end{array}$$

Using this free energy and the Boltzmann weight, we obtain the average of the endpoint position of the filament in the *x* coordinate in the fixed force (Gibbs) ensemble,

(28) $$\begin{array}{c} \langle x\rangle =\frac{\sum_{n=0}^{n=1}\int_{-\infty }^{+\infty }\left(x\right)\exp\left(-\frac{H_{x}^{\prime (n)}\left(L,l_{ps1},l_{ps2},\omega \right)}{k_{B}T}\right)dx}{\sum_{n=0}^{n=1}\int_{-\infty }^{+\infty }\exp\left(-\frac{H_{x}^{\prime (n)}\left(L,l_{ps1},l_{ps2},\omega \right)}{k_{B}T}\right)dx}\;. \end{array}$$

The result of the integrations in the above equation gives the force-extension relation in the *x* direction in the fixed force ensemble

(29) $$\begin{array}{c} \langle x-L\cos(\omega )\rangle =\frac{A_{x1}+A_{x2}}{B_{x1}+B_{x2}}\;. \end{array}$$

where

(30) $$A_{x1}=\frac{4\sqrt{3\pi }{\tilde{f}}_{x}}{9\sqrt{\frac{{l_{\mathrm{ps}1}}^{3}}{{\left(\sin\left(\omega \right)\right)}^{6}L^{9}}}}\exp(\frac{{{\tilde{f}}_{x}}^{2}{\left(\sin\left(\omega \right)\right)}^{2}L^{3}}{3l_{\mathrm{ps}1}})$$

and

(31) $$A_{x2}=\frac{4\sqrt{3\pi }{\tilde{f}}_{x}}{9\sqrt{\frac{{l_{\mathrm{ps}2}}^{3}}{{\left(\sin\left(\omega \right)\right)}^{6}L^{9}}}}\exp(\frac{{{\tilde{f}}_{x}}^{2}{\left(\sin\left(\omega \right)\right)}^{2}L^{3}}{3l_{\mathrm{ps}2}}-\tilde{\epsilon })$$

and

(32) $$B_{x1}=\frac{2\sqrt{3\pi }}{3\sqrt{\frac{l_{\mathrm{ps}1}}{{\left(\sin\left(\omega \right)\right)}^{2}L^{3}}}}\exp(\frac{{{\tilde{f}}_{x}}^{2}{\left(\sin\left(\omega \right)\right)}^{2}L^{3}}{3l_{\mathrm{ps}1}})$$

and

(33) $$B_{x2}=\frac{2\sqrt{3\pi }}{3\sqrt{\frac{l_{\mathrm{ps}2}}{{\left(\sin\left(\omega \right)\right)}^{2}L^{3}}}}\exp(\frac{{{\tilde{f}}_{x}}^{2}{\left(\sin\left(\omega \right)\right)}^{2}L^{3}}{3l_{\mathrm{ps}2}}-\tilde{\epsilon })$$

The average of *n* when the fixed force is exerted in the *x* direction is given by the following expression:

(34) $$\begin{array}{c} {\langle n\rangle }_{x}=\frac{\sum_{n=0}^{n=1}\int_{-\infty }^{+\infty }\left(n\right)\exp\left(-\frac{H_{x}^{\prime (n)}\left(L,l_{ps1},l_{ps2},\omega \right)}{k_{B}T}\right)dx}{\sum_{n=0}^{n=1}\int_{-\infty }^{+\infty }\exp\left(-\frac{H_{x}^{\prime (n)}\left(L,l_{ps1},l_{ps2},\omega \right)}{k_{B}T}\right)dx}\;. \end{array}$$

This leads to the following analytic expression for the average of *n*:

(35) $$\begin{array}{c} {\langle n\rangle }_{x}=\frac{e^{\frac{{\tilde{f_{x}}}^{2}L^{3}{\left(\sin\left(\omega \right)\right)}^{2}-3\;\tilde{\epsilon }\;l_{\mathrm{ps}2}}{3l_{\mathrm{ps}2}}}\sqrt{l_{\mathrm{ps}1}}}{A_{nx}} \end{array}$$

where

(36) $$\begin{array}{c} A_{nx}=e^{\frac{{\tilde{f_{x}}}^{2}L^{3}{\left(\sin\left(\omega \right)\right)}^{2}}{3l_{\mathrm{ps}1}}}\sqrt{l_{\mathrm{ps}2}}+e^{\frac{{\tilde{f_{x}}}^{2}L^{3}{\left(\sin\left(\omega \right)\right)}^{2}-3\;\tilde{\epsilon }\;l_{\mathrm{ps}2}}{3l_{\mathrm{ps}2}}}\sqrt{l_{\mathrm{ps}1}} \end{array}$$

The physical meaning of 〈n〉 is the ensemble probability of finding the two-level filament in the state with the persistence length $l_{p}=l_{ps2}$.

Let us now consider the two-state filament in the fixed extension ensemble. The probability density of the position of the tip of the filament in the *x* coordinate is given by the addition rule of the probability theory,

(37) $$P_{FE}\left(x\right)=\frac{1}{N_{FE}}\left(e^{-\frac{3l_{\mathrm{ps}1}{\left(x-L\;\cos\left(\omega \right)\right)}^{2}}{4{\left(\sin\left(\omega \right)\right)}^{2}L^{3}}}+e^{-\frac{3l_{\mathrm{ps}2}{\left(x-L\;\cos\left(\omega \right)\right)}^{2}}{4{\left(\sin\left(\omega \right)\right)}^{2}L^{3}}-\tilde{\epsilon }}\right)$$

where $N_{FE}$ is the normalization factor,

(38) $$N_{FE}=\frac{2\sqrt{3\pi \;{\left(\sin\left(\omega \right)\right)}^{2}L^{3}}}{3}\left(\sqrt{\frac{1}{l_{\mathrm{ps}1}}}+\sqrt{\frac{1}{l_{\mathrm{ps}2}}}\exp\left(-\tilde{\epsilon }\right)\right)$$

The Helmholtz free energy in the fixed extension ensemble can be calculated by

(39) $$F_{FE}\left(x\right)=-k_{B}T\ln\left(P_{FE}\left(x\right)\right)\;.$$

Therefore, the average of the fluctuating force, $\langle f\rangle$, in the fixed extension ensemble, is obtained by taking the derivative of the free energy,

(40) $$\begin{array}{ccc} \langle f\rangle & = & \frac{\partial F_{FE}\left(x\right)}{\partial x}\\ & = & \frac{3k_{B}T\left(x-L\;\cos\left(\omega \right)\right)}{2{\left(\sin\left(\omega \right)\right)}^{2}L^{3}}\frac{\left(l_{ps1}e^{-\frac{3l_{\mathrm{ps}1}{\left(x-L\;\cos\left(\omega \right)\right)}^{2}}{4{\left(\sin\left(\omega \right)\right)}^{2}L^{3}}}+l_{ps2}e^{-\frac{3l_{\mathrm{ps}2}{\left(x-L\;\cos\left(\omega \right)\right)}^{2}}{4{\left(\sin\left(\omega \right)\right)}^{2}L^{3}}-\tilde{\epsilon }}\right)}{\left(e^{-\frac{3l_{\mathrm{ps}1}{\left(x-L\;\cos\left(\omega \right)\right)}^{2}}{4{\left(\sin\left(\omega \right)\right)}^{2}L^{3}}}+e^{-\frac{3l_{\mathrm{ps}2}{\left(x-L\;\cos\left(\omega \right)\right)}^{2}}{4{\left(\sin\left(\omega \right)\right)}^{2}L^{3}}-\tilde{\epsilon }}\right)} \end{array}$$

This result is the force-extension relation in the fixed extension ensemble. We see that it differs from the corresponding force-extension relation in the Gibbs ensemble, Equation (29), thus implying ensemble inequivalence.

**Figure 3:**
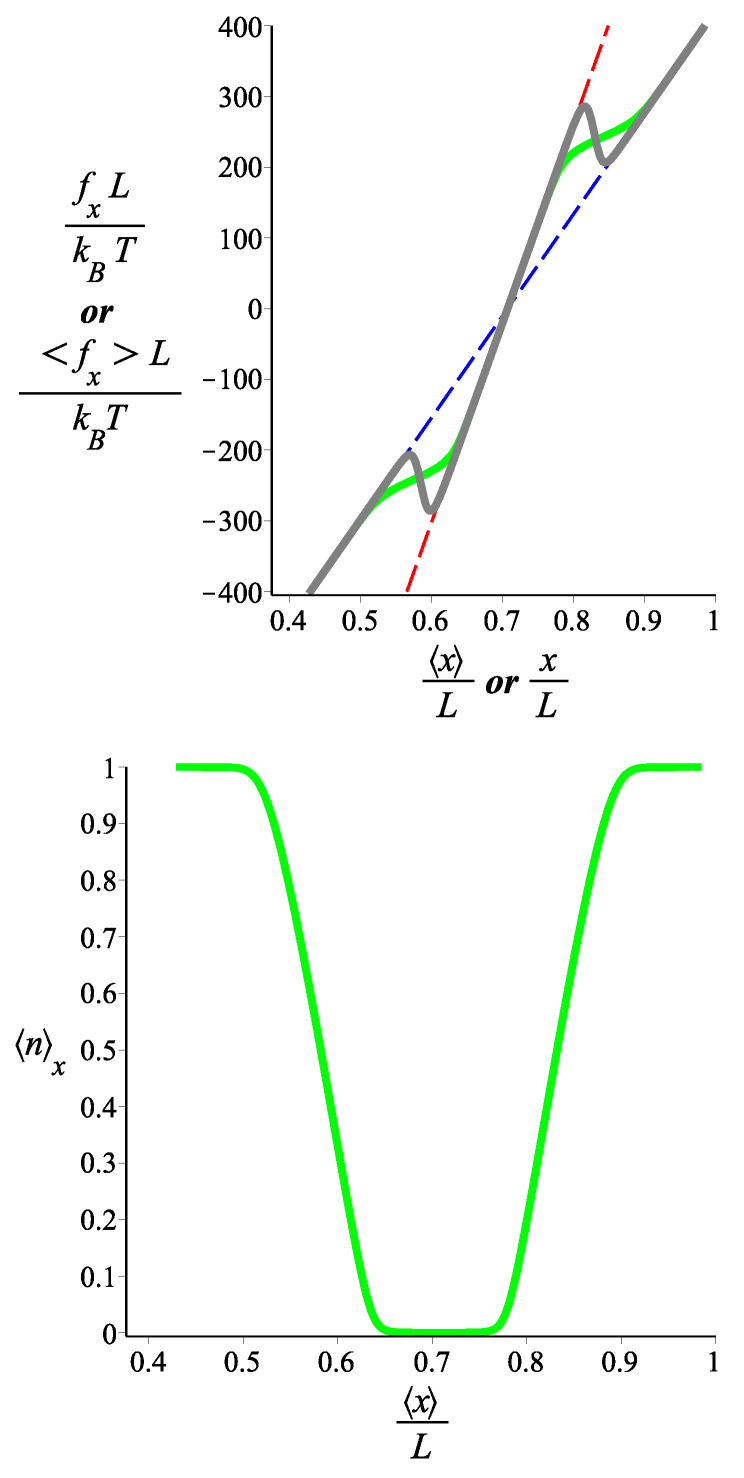
**Upper** panel: The force-extension relation of a single two-state filament in the Gibbs and the Helmholtz ensembles. The green curve corresponds to a single filament with fluctuating persistence length between the two values, $\frac{l_{ps1}}{L}=940$ and $\frac{l_{ps2}}{L}=480$ and it is given by Equation (29) (fixed force or Gibbs ensemble). The red and blue curves correspond to single filaments with fixed persistence lengths $l_{p}=l_{ps1}$ and $l_{p}=l_{ps2}$, respectively, which are given by Equation (9). The gray curve is the force-extension relation of the two-state filament in the fixed extension (Helmholtz) ensemble, given by Equation (40). **Lower** panel: The average of the state number in the Gibbs ensemble, 〈n〉, as a function of the average position in the *x* direction. It is calculated from the combination of Equation (35) and Equation (29). The fixed parameters are ω=π/4, $\tilde{\epsilon }=10$ for both panels. The contour length of the filament in the curves in both panels is the same and it is fixed: L=1.

In Figure 3, we see the force-extension relation in the two ensembles. For a small absolute value of the force (or, equivalently, for a small deformation), the filament is in the $l_{p}=l_{ps1}$ state. By increasing the absolute value of the force (or, equivalently, the deformation), the filament tends towards the $l_{p}=l_{ps2}$ state. The force-extension relation is monotonic in the Gibbs ensemble, but it becomes spiky with regions of negative compressibility and extensivity in the Helmholtz ensemble. In the lower panel of Figure 3, we see how the probability of occurrence of the $l_{p}=l_{ps2}$ state in the Gibbs ensemble varies as a function of the average *x* position of the filament tip. It becomes clear that the change in the stiffness of the filament follows the change in 〈n〉.

Now, we consider placing a stiff planar wall in front of the endpoint of the filament in the *x* direction which confines the tip of the filament in the *x* coordinate. The fractional change in the partition function due to the reduction of the number of configurational microstates associated with the tip of the filament induced by the presence of the confining wall in the *x* direction is given by the following expression:

(41) $$Z_{x}^{\left(n\right)}\left(\delta_{x}\right)=\frac{\sum_{n=0}^{n=1}\int_{-\infty }^{\delta_{x}}\exp\left(-\frac{H_{x}^{\left(n\right)}\left(L,l_{ps1},l_{ps2},\omega \right)}{k_{B}T}\right)dx}{\sum_{n=0}^{n=1}\int_{-\infty }^{+\infty }\exp\left(-\frac{H_{x}^{\left(n\right)}\left(L,l_{ps1},l_{ps2},\omega \right)}{k_{B}T}\right)dx}$$

We have to clarify that the superscript (n) in the above partition function does not imply a dependence on *n* (which is traced over), but it is meant to indicate that this partition function refers to a two-state filament instead of a filament with a fixed persistence length.

The average force on the wall is obtained by taking the derivative of the logarithm of the partition function in the *x* direction,

(42) $$\langle f_{x}^{PW}\rangle =k_{B}T\frac{d}{d\delta_{x}}\ln\left(Z_{x}^{\left(n\right)}\left(\delta_{x}\right)\right)$$

The result of the calculation gives the following closed analytic expression for the average of the force exerted by the tip of the filament on the wall:

(43) $$\begin{array}{cc} \frac{\langle f_{x}^{PW,n}\rangle }{k_{B}T} & =-\frac{\sqrt{3}\left(l_{\mathrm{ps}1}\sqrt{l_{\mathrm{ps}2}}+l_{\mathrm{ps}2}\sqrt{l_{\mathrm{ps}1}}e^{\tilde{\epsilon }}\right)e^{\tilde{\epsilon }}e^{\frac{3{\left(-\delta_{x}+L\cos\left(\omega \right)\right)}^{2}l_{\mathrm{ps}1}}{4{\left(\sin\left(\omega \right)\right)}^{2}L^{3}}}}{\sqrt{\pi }L^{3/2}\sin\left(\omega \right)\alpha }\\ & -\frac{\sqrt{3}\left(l_{\mathrm{ps}1}\sqrt{l_{\mathrm{ps}2}}+l_{\mathrm{ps}2}\sqrt{l_{\mathrm{ps}1}}e^{\tilde{\epsilon }}\right)e^{-\frac{3{\left(-\delta_{x}+L\cos\left(\omega \right)\right)}^{2}l_{\mathrm{ps}2}}{4{\left(\sin\left(\omega \right)\right)}^{2}L^{3}}}}{\sqrt{\pi }L^{3/2}\sin\left(\omega \right)\alpha }\;, \end{array}$$

where

(44) $$\begin{array}{cc} \alpha & =\left(-l_{\mathrm{ps}2}{\left(e^{\tilde{\epsilon }}\right)}^{2}-l_{\mathrm{ps}1}-2\;e^{\tilde{\epsilon }}\sqrt{l_{\mathrm{ps}1}}\sqrt{l_{\mathrm{ps}2}}\right)\\ & +{\left(e^{\tilde{\epsilon }}\right)}^{2}l_{\mathrm{ps}2}\mathrm{erf}\left(\frac{\sqrt{3l_{\mathrm{ps}1}}\left(-\delta_{x}+L\cos\left(\omega \right)\right)}{2L^{3/2}\sin\left(\omega \right)}\right)\\ & +e^{\tilde{\epsilon }}\sqrt{l_{\mathrm{ps}1}}\sqrt{l_{\mathrm{ps}2}}\mathrm{erf}\left(\frac{\sqrt{3l_{\mathrm{ps}1}}\left(-\delta_{x}+L\cos\left(\omega \right)\right)}{2L^{3/2}\sin\left(\omega \right)}\right)\\ & +e^{\tilde{\epsilon }}\sqrt{l_{\mathrm{ps}1}}\sqrt{l_{\mathrm{ps}2}}\mathrm{erf}\left(\frac{\sqrt{3l_{\mathrm{ps}2}}\left(-\delta_{x}+L\cos\left(\omega \right)\right)}{2L^{3/2}\sin\left(\omega \right)}\right)\\ & +l_{\mathrm{ps}1}\mathrm{erf}\left(\frac{\sqrt{3l_{\mathrm{ps}2}}\left(-\delta_{x}+L\cos\left(\omega \right)\right)}{2L^{3/2}\sin\left(\omega \right)}\right)\;. \end{array}$$

Meanwhile, the average of *n* for the case of the restricting wall in the *x* coordinate can be obtained as follows:

(45) $${\langle n\rangle }_{x}^{PW}=\frac{\sum_{n=0}^{n=1}\int_{-\infty }^{\delta_{x}}\left(n\right)\exp\left(-\frac{H_{x}^{\left(n\right)}\left(L,l_{ps1},l_{ps2},\omega \right)}{k_{B}T}\right)dx}{\sum_{n=0}^{n=1}\int_{-\infty }^{+\delta_{x}}\exp\left(-\frac{H_{x}^{\left(n\right)}\left(L,l_{ps1},l_{ps2},\omega \right)}{k_{B}T}\right)dx}\;.$$

The result of the integration in the above equation gives an analytic expression for the average *n* in this case,

(46) $$\begin{array}{c} {\langle n\rangle }_{x}^{PW}=\frac{\left(C_{x}^{PW}\left(l_{\mathrm{ps}2}\right)\right)\sqrt{l_{\mathrm{ps}1}}}{\left(C_{x}^{PW}\left(l_{\mathrm{ps}2}\right)\right)\sqrt{l_{\mathrm{ps}1}}+e^{\tilde{\epsilon }}\sqrt{l_{\mathrm{ps}2}}\left(C_{x}^{PW}\left(l_{\mathrm{ps}1}\right)\right)} \end{array}$$

where

(47) $$\begin{array}{c} C_{x}^{PW}\left(x\right)=\mathrm{erf}\left(\frac{\sqrt{3x}\left(-\delta_{x}+L\cos\left(\omega \right)\right)}{2L^{3/2}\sin\left(\omega \right)}\right)-1 \end{array}$$

**Figure 4:**
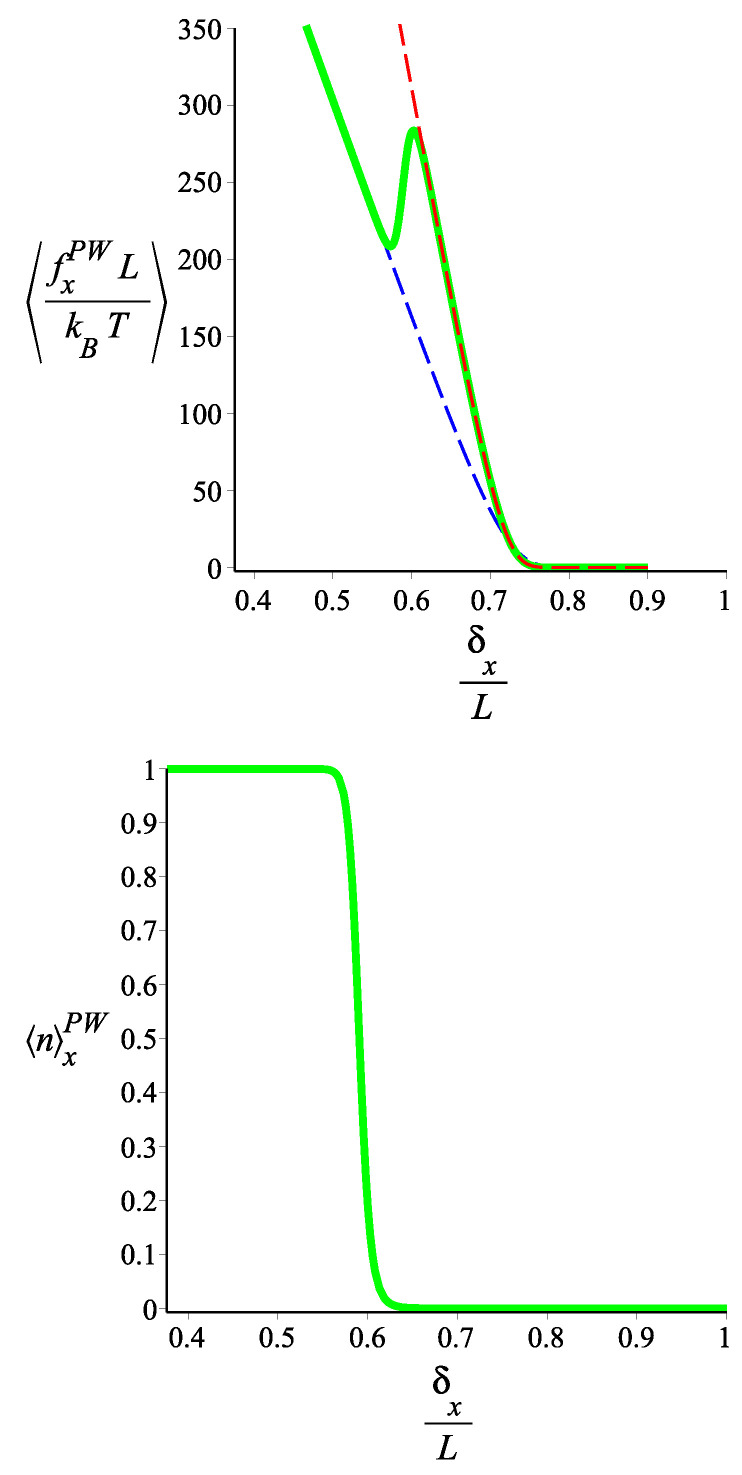
**Upper** panel: the average of the force exerted by the tip of the single-block two-state filament on the restricting stiff planar wall as a function of the position of the wall in the *x* coordinate. The green curve is the force-compression curve associated with a filament with fluctuating persistence length between the two values, $\frac{l_{ps1}}{L}=940$ and $\frac{l_{ps2}}{L}=480$ and, it is given by Equation (43). The red and the blue dashed curves are associated with single filaments with fixed persistence length $l_{p}=l_{ps1}$ and $l_{p}=l_{ps2}$, respectively, and are given by Equation (13). **Lower** panel: the average of the state number, *n* associated with a filament with persistence length fluctuating between the two values, $\frac{l_{ps1}}{L}=940$ and $\frac{l_{ps2}}{L}=480$, is shown as a function of the dimensionless position of the confining wall. It is plotted from Equation (46). The fixed parameters for all the curves in the upper panel and the lower panel are ω=π/4, $\tilde{\epsilon }=10$. The length of the filaments in all the cases is the same L=1.

In Figure 4, we see the force-compression curve for a single-block two-state grafted filament confined by a rigid planar wall in the *x* direction. For very small compression, the filament responds as if it had the persistence length $l_{p}=l_{ps1}$. As the compression increases, there is a transition to the $l_{p}=l_{ps2}$ state. This transition is also shown in the lower panel, where we plot the average value of the state parameter *n* as a function of the position of the wall. In the transition region, the filament exhibits negative compressibility. We point out that, since the wall is held at a fixed position, the response is in the Helmholtz ensemble.

In the remainder of this section, we consider forces and deformations of the two-state grafted filament in the *y* direction. In a similar fashion as in the analysis of the *x* direction, the Helmholtz free energy associated with the position of the tip of the filament in the *y* coordinate, for a given value of *n*, is given by

(48) $$\begin{array}{cc} H_{y}^{\left(n\right)}\left(L,l_{ps1},l_{ps2},\omega \right) & =H_{y}\left(L,l_{ps1}+n\left(l_{ps2}-l_{ps1}\right),\omega \right)\\ & +n\epsilon \\ & =\left(\frac{3k_{B}T(l_{ps1}+n\left(l_{ps2}-l_{ps1}\right){\left(y-L\sin\left(\omega \right)\right)}^{2}}{4L^{3}\cos^{2}\left(\omega \right)}\right)\\ & +n\epsilon \end{array}$$

The free energy in the presence of a fixed external force in the *y* direction (Gibbs free energy), $f_{y}\equiv k_{B}T\tilde{f_{y}}$ takes the following form of an effective Hamiltonian:

(49) $$\begin{array}{cc} H_{y}^{\prime (n)}\left(L,l_{ps1},l_{ps2},\omega \right) & =H_{y}^{\prime }\left(L,l_{ps1}+n\left(l_{ps2}-l_{ps1}\right),\omega \right)\\ & +n\epsilon \\ & =\left(\frac{3k_{B}T(l_{ps1}+n\left(l_{ps2}-l_{ps1}\right){\left(y-L\sin\left(\omega \right)\right)}^{2}}{4L^{3}\cos^{2}\left(\omega \right)}\right)\\ & -f_{y}\left(y-L\sin\left(\omega \right)\right)+n\epsilon \end{array}$$

Using the Boltzmann weight in the Gibbs ensemble, the average of the *y* component of the endpoint position of the filament for a fixed force is given by the following expression:

(50) $$\begin{array}{c} \langle y\rangle =\frac{\sum_{n=0}^{n=1}\int_{-\infty }^{+\infty }\left(y\right)\exp\left(-\frac{H_{y}^{\prime (n)}\left(L,l_{ps1},l_{ps2},\omega \right)}{k_{B}T}\right)dy}{\sum_{n=0}^{n=1}\int_{-\infty }^{+\infty }\exp\left(-\frac{H_{y}^{\prime (n)}\left(L,l_{ps1},l_{ps2},\omega \right)}{k_{B}T}\right)dy}\;. \end{array}$$

The above expression gives the force-extension relation in the *y* coordinate in the fixed force ensemble,

(51) $$\begin{array}{c} \langle y-L\sin(\omega )\rangle =\frac{A_{y1}+A_{y2}}{B_{y1}+B_{y2}}\;, \end{array}$$

where

(52) $$A_{y1}=\frac{4\sqrt{3\pi }{\tilde{f}}_{y}}{9\sqrt{\frac{{l_{\mathrm{ps}1}}^{3}}{{\left(\cos\left(\omega \right)\right)}^{6}L^{9}}}}e^{\frac{{{\tilde{f}}_{y}}^{2}{\left(\cos\left(\omega \right)\right)}^{2}L^{3}}{3l_{\mathrm{ps}1}}}$$

and

(53) $$A_{y2}=\frac{4\sqrt{3\pi }{\tilde{f}}_{y}}{9\sqrt{\frac{{l_{\mathrm{ps}2}}^{3}}{{\left(\cos\left(\omega \right)\right)}^{6}L^{9}}}}e^{\frac{{{\tilde{f}}_{y}}^{2}{\left(\cos\left(\omega \right)\right)}^{2}L^{3}}{3l_{\mathrm{ps}2}}-\tilde{\epsilon }}$$

and

(54) $$B_{y1}=\frac{2\sqrt{3\pi }}{3\sqrt{\frac{l_{\mathrm{ps}1}}{{\left(\cos\left(\omega \right)\right)}^{2}L^{3}}}}e^{\frac{{{\tilde{f}}_{y}}^{2}{\left(\cos\left(\omega \right)\right)}^{2}L^{3}}{3l_{\mathrm{ps}1}}}$$

and

(55) $$B_{y2}=\frac{2\sqrt{3\pi }}{3\sqrt{\frac{l_{\mathrm{ps}2}}{{\left(\cos\left(\omega \right)\right)}^{2}L^{3}}}}e^{\frac{{{\tilde{f}}_{y}}^{2}{\left(\cos\left(\omega \right)\right)}^{2}L^{3}}{3l_{\mathrm{ps}2}}-\tilde{\epsilon }}$$

The average of *n* when the force is exerted in the *y* direction is given by the following expression:

(56) $$\begin{array}{c} {\langle n\rangle }_{y}=\frac{\sum_{n=0}^{n=1}\int_{-\infty }^{+\infty }\left(n\right)\exp\left(-\frac{H_{y}^{\prime (n)}\left(L,l_{ps1},l_{ps2},\omega \right)}{k_{B}T}\right)dy}{\sum_{n=0}^{n=1}\int_{-\infty }^{+\infty }\exp\left(-\frac{H_{y}^{\prime (n)}\left(L,l_{ps1},l_{ps2},\omega \right)}{k_{B}T}\right)dy}\;. \end{array}$$

We point out that this is a result pertaining to the Gibbs ensemble, so it depends on the force and not on the position. The subscript *y* simply indicates the direction. The result of the integration is the following expression for the average of *n* in the case of the presence of a fixed external force in the *y* direction:

(57) $$\begin{array}{c} {\langle n\rangle }_{y}=\frac{e^{\frac{{\tilde{f_{y}}}^{2}L^{3}{\left(\cos\left(\omega \right)\right)}^{2}-3\;\tilde{\epsilon }\;l_{\mathrm{ps}2}}{3l_{\mathrm{ps}2}}}\sqrt{l_{\mathrm{ps}1}}}{A_{ny}}\;, \end{array}$$

where

(58) $$\begin{array}{c} A_{ny}=e^{\frac{{\tilde{f_{y}}}^{2}L^{3}{\left(\cos\left(\omega \right)\right)}^{2}}{3l_{\mathrm{ps}1}}}\sqrt{l_{\mathrm{ps}2}}+e^{\frac{{\tilde{f_{y}}}^{2}L^{3}{\left(\cos\left(\omega \right)\right)}^{2}-3\;\tilde{\epsilon }\;l_{\mathrm{ps}2}}{3l_{\mathrm{ps}2}}}\sqrt{l_{\mathrm{ps}1}}\;. \end{array}$$

Now, we put a stiff planar wall in front of the tip of the filament of confine it in the *y* direction. The fractional (relative) change in the partition function due to the reduction of the number of configurations of the tip of the filament in the presence of the wall has the following form:

(59) $$Z_{y}^{\left(n\right)}\left(\delta_{y}\right)=\frac{\sum_{n=0}^{n=1}\int_{-\infty }^{\delta_{y}}\exp\left(-\frac{H_{y}^{\left(n\right)}\left(L,l_{ps1},l_{ps2},\omega \right)}{k_{B}T}\right)dy}{\sum_{n=0}^{n=1}\int_{-\infty }^{+\infty }\exp\left(-\frac{H_{y}^{\left(n\right)}\left(L,l_{ps1},l_{ps2},\omega \right)}{k_{B}T}\right)dy}$$

The average of the *y* component of the force on the wall is given by the derivative of the free energy associated with the tip of the filament in the *y* direction,

(60) $$\langle f_{y}^{PW,n}\rangle =k_{B}T\frac{d}{d\delta_{y}}\ln\left(Z_{y}^{\left(n\right)}\left(\delta_{y}\right)\right)$$

The result is a closed expression for the average of the *y* component of the force on the wall,

(61) $$\begin{array}{cc} \frac{\langle f_{y}^{PW,n}\rangle }{k_{B}T} & =-\frac{\sqrt{3}\left(l_{\mathrm{ps}1}\sqrt{l_{\mathrm{ps}2}}+l_{\mathrm{ps}2}\sqrt{l_{\mathrm{ps}1}}e^{\tilde{\epsilon }}\right)e^{\tilde{\epsilon }}e^{\frac{3{\left(-\delta_{y}+L\sin\left(\omega \right)\right)}^{2}l_{\mathrm{ps}1}}{4{\left(\cos\left(\omega \right)\right)}^{2}L^{3}}}}{\sqrt{\pi }L^{3/2}\cos\left(\omega \right)\alpha }\\ & -\frac{\sqrt{3}\left(l_{\mathrm{ps}1}\sqrt{l_{\mathrm{ps}2}}+l_{\mathrm{ps}2}\sqrt{l_{\mathrm{ps}1}}e^{\tilde{\epsilon }}\right)e^{-\frac{3{\left(-\delta_{y}+L\sin\left(\omega \right)\right)}^{2}l_{\mathrm{ps}2}}{4{\left(\cos\left(\omega \right)\right)}^{2}L^{3}}}}{\sqrt{\pi }L^{3/2}\cos\left(\omega \right)\alpha } \end{array}$$

where

(62) $$\begin{array}{cc} \alpha & =\left(-l_{\mathrm{ps}2}{\left(e^{\tilde{\epsilon }}\right)}^{2}-l_{\mathrm{ps}1}-2\;e^{\tilde{\epsilon }}\sqrt{l_{\mathrm{ps}1}}\sqrt{l_{\mathrm{ps}2}}\right)\\ & +{\left(e^{\tilde{\epsilon }}\right)}^{2}l_{\mathrm{ps}2}\mathrm{erf}\left(\frac{\sqrt{3l_{\mathrm{ps}1}}\left(-\delta_{y}+L\sin\left(\omega \right)\right)}{2L^{3/2}\cos\left(\omega \right)}\right)\\ & +e^{\tilde{\epsilon }}\sqrt{l_{\mathrm{ps}1}}\sqrt{l_{\mathrm{ps}2}}\mathrm{erf}\left(\frac{\sqrt{3l_{\mathrm{ps}1}}\left(-\delta_{y}+L\sin\left(\omega \right)\right)}{2L^{3/2}\cos\left(\omega \right)}\right)\\ & +e^{\tilde{\epsilon }}\sqrt{l_{\mathrm{ps}1}}\sqrt{l_{\mathrm{ps}2}}\mathrm{erf}\left(\frac{\sqrt{3l_{\mathrm{ps}2}}\left(-\delta_{y}+L\sin\left(\omega \right)\right)}{2L^{3/2}\cos\left(\omega \right)}\right)\\ & +l_{\mathrm{ps}1}\mathrm{erf}\left(\frac{\sqrt{3l_{\mathrm{ps}2}}\left(-\delta_{y}+L\sin\left(\omega \right)\right)}{2L^{3/2}\cos\left(\omega \right)}\right)\;. \end{array}$$

We point out that since the position of the wall is fixed, this is a result in the Gibbs ensemble.

Also, we can calculate the average of *n* as a function of the distance of the wall from the grafting point in the *y* direction using the Boltzmann weight in the canonical (Gibbs) ensemble,

(63) $${\langle n\rangle }_{y}^{PW}=\frac{\sum_{n=0}^{n=1}\int_{-\infty }^{\delta_{y}}\left(n\right)\exp\left(-\frac{H_{y}^{\left(n\right)}\left(L,l_{ps1},l_{ps2},\omega \right)}{k_{B}T}\right)dy}{\sum_{n=0}^{n=1}\int_{-\infty }^{+\delta_{y}}\exp\left(-\frac{H_{y}^{\left(n\right)}\left(L,l_{ps1},l_{ps2},\omega \right)}{k_{B}T}\right)dy}$$

The integrations in the above equation lead to the analytic expression for the average of *n* in the case in which the wall restricts the tip of the filament in the *y* direction,

(64) $$\begin{array}{c} {\langle n\rangle }_{y}^{PW}=\frac{\left(C_{y}^{PW}\left(l_{\mathrm{ps}2}\right)\right)\sqrt{l_{\mathrm{ps}1}}}{\left(C_{y}^{PW}\left(l_{\mathrm{ps}2}\right)\right)\sqrt{l_{\mathrm{ps}1}}+e^{\tilde{\epsilon }}\sqrt{l_{\mathrm{ps}2}}\left(C_{y}^{PW}\left(l_{\mathrm{ps}1}\right)\right)}\;, \end{array}$$

where

(65) $$\begin{array}{c} C_{x}^{PW}\left(x\right)=\mathrm{erf}\left(\frac{\sqrt{3x}\left(-\delta +L\sin\left(\omega \right)\right)}{2L^{3/2}\cos\left(\omega \right)}\right)-1\;. \end{array}$$

## 4. Grafted Filament with Two Blocks, Each Having a Fixed Persistence Length

**Figure 5:**
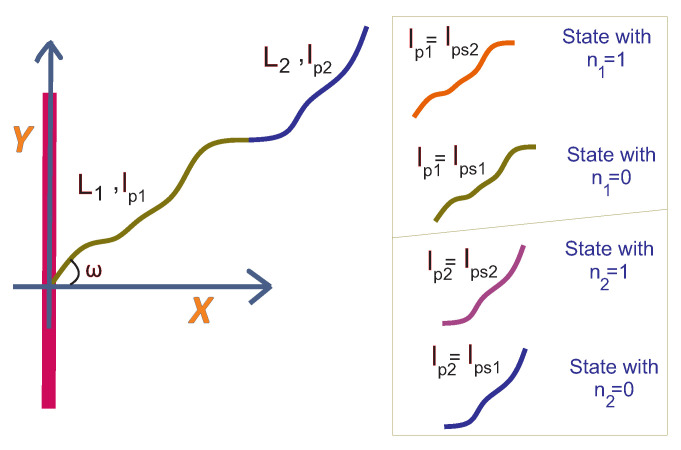
The grafted filament with two blocks of persistence lengths $l_{p1},l_{p2}$ and of contour lengths $L_{1},L_{2}$ at a finite temperature. In the case of a filament with fluctuating persistence lengths, the persistence length of the first block can fluctuate between two states with the state number $n_{1}\in \left\{0,1\right\}$ which are associated with two values for the persistence length $l_{p1}\in \left\{l_{ps1},l_{ps2}\right\}$, and the persistence length of the second block can fluctuate between two other independent states with state number $n_{2}\in \left\{0,1\right\}$ which are associated with the two values of the persistence length $l_{p2}\in \left\{l_{ps1}^{\prime },l_{ps2}^{\prime }\right\}$. The transition from the state, $n_{1}=0$ to state, $n_{1}=1$ requires a fixed energetic cost denoted by $\epsilon_{1}=k_{B}T\tilde{\epsilon_{1}}$. Also, the transition from the state $n_{2}=0$ to state $n_{2}=1$ requires another fixed energetic cost of $\epsilon_{2}=k_{B}T\tilde{\epsilon_{2}}$. The total contour length of the filament is denoted by $L=L_{1}+L_{2}$.

The Hamiltonian of a wormlike chain consisting of two concatenated blocks with two different persistence lengths such as $l_{p1}$ and $l_{p2}$ and two different contour lengths $L_{1}$ and $L_{2}$ is as follows (see Figure 5):

(66) $$\begin{array}{c} H_{WLC}^{2b}=\frac{k_{B}Tl_{p1}}{4}\int_{0}^{L_{1}}{\left(\frac{dt(s)}{ds}\right)}^{2}ds+\frac{k_{B}Tl_{p2}}{4}\int_{L_{1}}^{L}{\left(\frac{dt(s)}{ds}\right)}^{2}ds\;, \end{array}$$

where $L=L_{1}+L_{2}$. The probability density of the endpoint position of the filament in the *x* direction can be obtained by concatenating two propagators,

(67) $$\begin{array}{cc} P_{x}^{2b}\left(x_{2}\right) & =\int \int dy_{1}dx_{1}d\theta_{1}dy_{2}d\theta_{2}\\ & \times G_{L_{1},l_{p1}}\left(x_{1},y_{1},\theta_{1}|0,0,\omega \right)\\ & \times G_{L_{2},l_{p2}}\left(x_{2},y_{2},\theta_{2}|x_{1},y_{1},\theta_{1}\right)\\ & \propto \exp\left(-\frac{{\left(x_{2}-\left(L_{1}+L_{2}\right)\cos\left(\omega \right)\right)}^{2}}{\sigma_{x}\left(L_{1},L_{2},l_{p1},l_{p2},\omega \right)}\right)\;, \end{array}$$

where

(68) $$\begin{array}{cc} \sigma_{x}\left(L_{1},L_{2},l_{p1},l_{p2},\omega \right) & =\frac{4\left(3\;{L_{2}}^{2}l_{p2}L_{1}+l_{p2}{L_{1}}^{3}\right)}{3l_{p2}l_{p1}}{\left(\sin\left(\omega \right)\right)}^{2}\\ & +\frac{4\left(3\;{L_{1}}^{2}L_{2}l_{p2}+l_{p1}{L_{2}}^{3}\right)}{3l_{p2}l_{p1}}{\left(\sin\left(\omega \right)\right)}^{2}\;. \end{array}$$

It is useful to mention that Equation (67) is special case of Equation (18) in Ref. [38] when the kink angle is fixed to zero. The probability density implies the following Helmholtz free energy in the *x* coordinate:

(69) $$H_{x}^{2b}\left(L_{1},L_{2},l_{p1},l_{p2},\omega \right)=\frac{k_{B}T{\left(x_{2}-\left(L_{1}+L_{2}\right)\cos\left(\omega \right)\right)}^{2}}{\sigma_{x}\left(L_{1},L_{2},l_{p1},l_{p2},\omega \right)}$$

The corresponding free energy in the presence of a fixed external force in the *x* direction takes the form of an effective Hamiltonian as

(70) $$\begin{array}{cc} H_{x}^{\prime 2b}\left(L_{1},L_{2},l_{p1},l_{p2},\omega \right) & =\frac{k_{B}T{\left(x_{2}-\left(L_{1}+L_{2}\right)\cos\left(\omega \right)\right)}^{2}}{\sigma_{x}\left(L_{1},L_{2},l_{p1},l_{p2},\omega \right)}\\ & -f_{x}\left(x_{2}-\left(L_{1}+L_{2}\right)\cos\left(\omega \right)\right)\;. \end{array}$$

The average *x* position of the tip in the fixed force (Gibbs) ensemble is given by the following integrations:

(71) $$\begin{array}{c} \langle x_{2}\rangle =\frac{\int_{-\infty }^{+\infty }\left(x_{2}\right)\exp\left(-\frac{H_{x}^{\prime 2b}\left(L_{1},L_{2},l_{p1},l_{p2},\omega \right)}{k_{B}T}\right)dx_{2}}{\int_{-\infty }^{+\infty }\exp\left(-\frac{H_{x}^{\prime 2b}\left(L_{1},L_{2},l_{p1},l_{p2},\omega \right)}{k_{B}T}\right)dx_{2}} \end{array}$$

The resulting force-extension relation in the *x* coordinate in the fixed force ensemble is

(72) $$\begin{array}{c} f_{x}=\frac{2k_{B}T\left(\langle x_{2}\rangle -\left(L_{1}+L_{2}\right)\cos\left(\omega \right)\right)}{\sigma_{x}\left(L_{1},L_{2},l_{p1},l_{p2},\omega \right)}\;. \end{array}$$

Similar to the previous sections, the force exerted by the tip of the filament to a confining wall in the *x* direction is given by the following expression:

(73) $$\begin{array}{c} \langle f_{x}^{PW,2b}\rangle =k_{B}T\frac{d}{d\delta_{x}}\ln\left(Z_{x}^{2b}\left(\delta_{x}\right)\right)\;, \end{array}$$

where

(74) $$\begin{array}{c} Z_{x}^{2b}\left(\delta_{x}\right)=\frac{\int_{-\infty }^{\delta_{x}}\exp\left(\frac{-H_{x}^{2b}\left(L_{1},L_{2},l_{p1},l_{p2},\omega \right)}{k_{B}T}\right)dx_{2}}{\int_{-\infty }^{+\infty }\exp\left(\frac{-H_{x}^{2b}\left(L_{1},L_{2},l_{p1},l_{p2},\omega \right)}{k_{B}T}\right)dx_{2}}\;. \end{array}$$

After performing the calculations, we obtain the following analytic expression for the average of the force on the fixed wall:

(75) $$\begin{array}{c} \langle f_{x}^{PW,2b}\rangle =\frac{2k_{B}T\;e^{-\frac{{\left(\left(L_{1}+L_{2}\right)\cos\left(\omega \right)-\delta_{x}\right)}^{2}}{\sigma_{x}\left(L_{1},L_{2},l_{p1},l_{p2},\omega \right)}}}{\sqrt{\pi \sigma_{x}\left(L_{1},L_{2},l_{p1},l_{p2},\omega \right)\left({C_{x}}^{2b}\right)}}\;, \end{array}$$

where

(76) $$\begin{array}{c} {C_{x}}^{2b}\equiv -1+\mathrm{erf}\left(\frac{\left(L_{1}+L_{2}\right)\cos\left(\omega \right)-\delta_{x}}{\sqrt{\sigma_{x}\left(L_{1},L_{2},l_{p1},l_{p2},\omega \right)}}\right)\;. \end{array}$$

The probability density of the endpoint position of the filament in the *y* coordinate can be obtained using the propagator method,

(77) $$\begin{array}{cc} P_{y}^{2b}\left(y_{2}\right) & =\int \int dy_{1}dx_{1}d\theta_{1}dx_{2}d\theta_{2}\\ & \times G_{L_{1},l_{p1}}\left(x_{1},y_{1},\theta_{1}|0,0,\omega \right)\\ & \times G_{L_{2},l_{p2}}\left(x_{2},y_{2},\theta_{2}|x_{1},y_{1},\theta_{1}\right)\\ & \propto \exp\left(-\frac{{\left(y_{2}-\left(L_{1}+L_{2}\right)\sin\left(\omega \right)\right)}^{2}}{\sigma_{y}\left(L_{1},L_{2},l_{p1},l_{p2},\omega \right)}\right)\;, \end{array}$$

where

(78) $$\begin{array}{cc} \sigma_{y}\left(L_{1},L_{2},l_{p1},l_{p2},\omega \right) & =\frac{4\left(3\;{L_{2}}^{2}l_{p2}L_{1}+l_{p2}{L_{1}}^{3}\right)}{3l_{p2}l_{p1}}{\left(\cos\left(\omega \right)\right)}^{2}\\ & +\frac{4\left(3\;{L_{1}}^{2}L_{2}l_{p2}+l_{p1}{L_{2}}^{3}\right)}{3l_{p2}l_{p1}}{\left(\cos\left(\omega \right)\right)}^{2}\;. \end{array}$$

Similar to the case of Equation (67), Equation (77) is a special case of Equation (20) in Ref. [38] when the kink angle is fixed to zero. The probability density defines the following Helmholtz free energy in the *y* coordinate:

(79) $$H_{y}^{2b}\left(L_{1},L_{2},l_{p1},l_{p2},\omega \right)=\frac{k_{B}T{\left(y_{2}-\left(L_{1}+L_{2}\right)\sin\left(\omega \right)\right)}^{2}}{\sigma_{y}\left(L_{1},L_{2},l_{p1},l_{p2},\omega \right)}\;.$$

The free energy in the presence of a fixed external force in the *y* direction takes the following form of an effective Hamiltonian:

(80) $$\begin{array}{cc} H_{y}^{\prime 2b}\left(L_{1},L_{2},l_{p1},l_{p2},\omega \right) & =\frac{k_{B}T{\left(y_{2}-\left(L_{1}+L_{2}\right)\sin\left(\omega \right)\right)}^{2}}{\sigma_{y}\left(L_{1},L_{2},l_{p1},l_{p2},\omega \right)}\\ & -f_{y}\left(y_{2}-\left(L_{1}+L_{2}\right)\sin\left(\omega \right)\right)\;. \end{array}$$

The average *y* position of the tip in the fixed force ensemble is given by the following integrations:

(81) $$\begin{array}{c} \langle y_{2}\rangle =\frac{\int_{-\infty }^{+\infty }\left(y_{2}\right)\exp\left(-\frac{H_{y}^{\prime 2b}\left(L_{1},L_{2},l_{p1},l_{p2},\omega \right)}{k_{B}T}\right)dy_{2}}{\int_{-\infty }^{+\infty }\exp\left(-\frac{H_{y}^{\prime 2b}\left(L_{1},L_{2},l_{p1},l_{p2},\omega \right)}{k_{B}T}\right)dy_{2}}\;. \end{array}$$

The resulting force-extension relation in the *y* coordinate in the fixed force ensemble is

(82) $$\begin{array}{c} f_{y}=\frac{2k_{B}T\left(\langle y_{2}\rangle -\left(L_{1}+L_{2}\right)\sin\left(\omega \right)\right)}{\sigma_{y}\left(L_{1},L_{2},l_{p1},l_{p2},\omega \right)} \end{array}$$

The force exerted by the tip of the filament to a confining wall in the *y* direction is given by the following expression:

(83) $$\begin{array}{c} \langle f_{y}^{PW,2b}\rangle =k_{B}T\frac{d}{d\delta_{y}}\ln\left(Z_{y}^{2b}\left(\delta_{y}\right)\right)\;, \end{array}$$

where

(84) $$\begin{array}{c} Z_{y}^{2b}\left(\delta_{x}\right)=\frac{\int_{-\infty }^{\delta_{y}}\exp\left(\frac{-H_{y}^{2b}\left(L_{1},L_{2},l_{p1},l_{p2},\omega \right)}{k_{B}T}\right)dy_{2}}{\int_{-\infty }^{+\infty }\exp\left(\frac{-H_{y}^{2b}\left(L_{1},L_{2},l_{p1},l_{p2},\omega \right)}{k_{B}T}\right)dy_{2}}\;. \end{array}$$

After performing the calculations, we obtain the following analytic expression for the average of the force on the wall:

(85) $$\begin{array}{c} \langle f_{y}^{PW,2b}\rangle =\frac{2k_{B}T\;e^{-\frac{{\left(\left(L_{1}+L_{2}\right)\sin\left(\omega \right)-\delta_{y}\right)}^{2}}{\sigma_{y}\left(L_{1},L_{2},l_{p1},l_{p2},\omega \right)}}}{\sqrt{\pi \sigma_{y}\left(L_{1},L_{2},l_{p1},l_{p2},\omega \right)\left({C_{y}}^{2b}\right)}}\;, \end{array}$$

where

(86) $$\begin{array}{c} {C_{y}}^{2b}\equiv -1+\mathrm{erf}\left(\frac{\left(L_{1}+L_{2}\right)\sin\left(\omega \right)-\delta_{y}}{\sqrt{\sigma_{y}\left(L_{1},L_{2},l_{p1},l_{p2},\omega \right)}}\right)\;. \end{array}$$

## 5. Grafted Filament with Two Blocks with Fluctuating Persistence Lengths

In this section, we consider a grafted filament consisting of two two-state blocks (see Figure 5). The first block and the second one have contour lengths of $L_{1}$ and $L_{2}$, respectively. The persistence length of the first block fluctuates between two states denoted by the Ising variable $n_{1}=0,1$. The first state and the second one correspond to the two values of the persistence length, $l_{ps1}$ and $l_{ps2}$, respectively. The energy difference of the two states of the first block is $\epsilon_{1}=k_{B}T\tilde{\epsilon_{1}}$. Meanwhile, the persistence length of the second block independently fluctuates between the two states which are denoted by $n_{2}=0,1$. The first state and the second one are associated with the two values of the persistence lengths, $l_{ps1}^{\prime }$ and $l_{ps2}^{\prime }$, respectively. The energy difference between the two states is $\epsilon_{2}=k_{B}T\tilde{\epsilon_{2}}$. The bending Hamiltonian of such a filament can be written as follows:

(87) $$\begin{array}{cc} H_{WLC}^{\left(n_{1},n_{2}\right)} & =\frac{k_{B}T\left(l_{ps1}+n_{1}\left(l_{ps2}-l_{ps1}\right)\right)}{4}\int_{0}^{L_{1}}{\left(\frac{dt(s)}{ds}\right)}^{2}ds+n_{1}\epsilon_{1}\\ & +\frac{k_{B}T\left(l_{ps1}^{\prime }+n_{2}\left(l_{ps2}^{\prime }-l_{ps1}^{\prime }\right)\right)}{4}\int_{L_{1}}^{L}{\left(\frac{dt(s)}{ds}\right)}^{2}ds+n_{2}\epsilon_{2}\; \end{array}$$

where $L=L_{1}+L_{2}$. The above Hamiltonian leads to the following expression for the Helmholtz free energy of the tip of the filament in the *x* direction, for a given $\left(n_{1},n_{2}\right)$ state:

(88) $$\begin{array}{cc} H_{x}^{n_{1},n_{2}} & (L_{1},L_{2},l_{ps1}+n_{1}\left(l_{ps2}-l_{ps1}\right),l_{ps1}^{\prime }+n_{2}\left(l_{ps2}^{\prime }-l_{ps1}^{\prime }\right),\omega )=\\ & +\frac{k_{B}T{\left(x_{2}-\left(L_{1}+L_{2}\right)\cos\left(\omega \right)\right)}^{2}}{\sigma_{x}\left(L_{1},L_{2},l_{ps1}+n_{1}\left(l_{ps2}-l_{ps1}\right),l_{ps1}^{\prime }+n_{2}\left(l_{ps2}^{\prime }-l_{ps1}^{\prime }\right),\omega \right)}\\ & +n_{1}\epsilon_{1}+n_{2}\epsilon_{2}\;. \end{array}$$

Therefore, the free energy in the presence of a fixed external force in the *x* direction gets the following form of an effective Hamiltonian:

(89) $$\begin{array}{cc} H_{x}^{\prime n_{1},n_{2}} & (L_{1},L_{2},l_{ps1}+n_{1}\left(l_{ps2}-l_{ps1}\right),l_{ps1}^{\prime }+n_{2}\left(l_{ps2}^{\prime }-l_{ps1}^{\prime }\right),\omega )=\\ & +\frac{k_{B}T{\left(x_{2}-\left(L_{1}+L_{2}\right)\cos\left(\omega \right)\right)}^{2}}{\sigma_{x}\left(L_{1},L_{2},l_{ps1}+n_{1}\left(l_{ps2}-l_{ps1}\right),l_{ps1}^{\prime }+n_{2}\left(l_{ps2}^{\prime }-l_{ps1}^{\prime }\right),\omega \right)}\\ & -f_{x}\left(x_{2}-\left(L_{1}+L_{2}\right)\cos\left(\omega \right)\right)+n_{1}\epsilon_{1}+n_{2}\epsilon_{2} \end{array}$$

The average position of the tip in the *x* direction in the presence of a fixed force is given by the following expression:

(90) $$\begin{array}{c} \langle x_{2}\rangle =\frac{\sum_{n_{1}=0}^{n_{1}=1}\sum_{n_{2}=0}^{n_{2}=1}\int_{-\infty }^{+\infty }\left(x_{2}\right)\exp\left(-\frac{H_{x}^{\prime n_{1},n_{2}}}{k_{B}T}\right)dx_{2}}{\sum_{n_{1}=0}^{n_{1}=1}\sum_{n_{2}=0}^{n_{2}=1}\int_{-\infty }^{+\infty }\exp\left(-\frac{H_{x}^{\prime n_{1},n_{2}}}{k_{B}T}\right)dx_{2}}\;, \end{array}$$

where we use the following abbreviation:

(91) $$H_{x}^{\prime n_{1},n_{2}}\equiv H_{x}^{\prime n_{1},n_{2}}\left(L_{1},L_{2},l_{ps1}+n_{1}\left(l_{ps2}-l_{ps1}\right),l_{ps1}^{\prime }+n_{2}\left(l_{ps2}^{\prime }-l_{ps1}^{\prime }\right),\omega \right)\;.$$

We obtain the following analytic expression for the force-extension relation in the *x* direction (in the fixed force ensemble):

(92) $$\langle x_{2}\rangle -\left(L_{1}+L_{2}\right)\cos\left(\omega \right)=\frac{d_{x,1}}{d_{x,2}}\;,$$

where

(93) $$\begin{array}{c} d_{x,1}=\frac{\sqrt{\pi }\tilde{f_{x}}}{2}\sum_{n_{1}=0}^{n_{1}=1}\sum_{n_{2}=0}^{n_{2}=1}{{(\hat{\sigma^{x}}}_{n_{1},n_{2}})}^{3/2}e^{\left(-n_{1}\;{\tilde{\epsilon }}_{1}-n_{2}\;{\tilde{\epsilon }}_{2}+1/4\;{\tilde{f_{x}}}^{2}{\hat{\sigma^{x}}}_{n_{1},n_{2}}\right)} \end{array}$$

and

(94) $$\begin{array}{c} d_{x,2}=\sum_{n_{1}=0}^{n_{1}=1}\sum_{n_{2}=0}^{n_{2}=1}{\left(\pi {\hat{\sigma^{x}}}_{n_{1},n_{2}}\right)}^{\frac{1}{2}}e^{-n_{1}\;{\tilde{\epsilon }}_{1}-n_{2}\;{\tilde{\epsilon }}_{2}+1/4\;{\tilde{f_{x}}}^{2}{\hat{\sigma^{x}}}_{n_{1},n_{2}}} \end{array}$$

and ${\hat{\sigma^{x}}}_{n_{1},n_{2}}$ is a 2×2 matrix with the following elements:

(95) $${\hat{\sigma^{x}}}_{0,0}=\sigma_{x}\left(L_{1},L_{2},l_{ps1},l_{ps1}^{\prime },\omega \right)$$

$${\hat{\sigma^{x}}}_{0,1}=\sigma_{x}\left(L_{1},L_{2},l_{ps1},l_{ps2}^{\prime },\omega \right)$$

$${\hat{\sigma^{x}}}_{1,0}=\sigma_{x}\left(L_{1},L_{2},l_{ps2},l_{ps1}^{\prime },\omega \right)$$

$${\hat{\sigma^{x}}}_{1,1}=\sigma_{x}\left(L_{1},L_{2},l_{ps2},l_{ps2}^{\prime },\omega \right)$$

The average of the state occupation numbers $n_{1}$, and $n_{2}$ in the fixed force ensemble in the *x* coordinate can be obtained as follows:

(96) $$\begin{array}{c} {\langle n_{i}\rangle }_{x_{2}}=\frac{\sum_{n_{1}=0}^{n_{1}=1}\sum_{n_{2}=0}^{n_{2}=1}\int_{-\infty }^{+\infty }\left(n_{i}\right)\exp\left(-\frac{H_{x}^{\prime n_{1},n_{2}}}{k_{B}T}\right)dx_{2}}{\sum_{n_{1}=0}^{n_{1}=1}\sum_{n_{2}=0}^{n_{2}=1}\int_{-\infty }^{+\infty }\exp\left(-\frac{H_{x}^{\prime n_{1},n_{2}}}{k_{B}T}\right)dx_{2}}\;. \end{array}$$

The result of the integration gives the following closed analytic expression for the average of the state occupation numbers:

(97) $$\begin{array}{c} {\langle n_{i}\rangle }_{x_{2}}=\frac{\sum_{n_{1}=0}^{n_{1}=1}\sum_{n_{2}=0}^{n_{2}=1}\left(\left(n_{i}\right)e^{-n_{1}\;{\tilde{\epsilon }}_{1}-n_{2}\;{\tilde{\epsilon }}_{2}+1/4\;{\tilde{f_{x}}}^{2}{\hat{\sigma^{x}}}_{n_{1},n_{2}}}\sqrt{{\hat{\sigma^{x}}}_{n_{1},n_{2}}}\right)}{\sum_{n_{1}=0}^{n_{1}=1}\sum_{n_{2}=0}^{n_{2}=1}\left(e^{-n_{1}\;{\tilde{\epsilon }}_{1}-n_{2}\;{\tilde{\epsilon }}_{2}+1/4\;{\tilde{f_{x}}}^{2}{\hat{\sigma^{x}}}_{n_{1},n_{2}}}\sqrt{{\hat{\sigma^{x}}}_{n_{1},n_{2}}}\right)}\;. \end{array}$$

The force exerted by the tip of the filament to a confining wall in the *x* direction is given by the following expression:

(98) $$\begin{array}{c} \langle f_{x}^{PW,n_{1},n_{2}}\rangle =k_{B}T\frac{d}{d\delta_{x}}\ln\left(Z_{x}^{n_{1},n_{2}}\left(\delta_{x}\right)\right)\;, \end{array}$$

where $\delta_{x}$ is the distance of the wall from the grafting point in the *x* direction and

(99) $$\begin{array}{c} Z_{x}^{n_{1},n_{2}}\left(\delta_{x}\right)=\frac{\sum_{n_{1}=0}^{n_{1}=1}\sum_{n_{2}=0}^{n_{2}=1}\int_{-\infty }^{\delta_{x}}\exp\left(\frac{-H_{x}^{n_{1},n_{2}}\left(L_{1},L_{2},l_{p1},l_{p2},\omega \right)}{k_{B}T}\right)dx_{2}}{\sum_{n_{1}=0}^{n_{1}=1}\sum_{n_{2}=0}^{n_{2}=1}\int_{-\infty }^{+\infty }\exp\left(\frac{-H_{x}^{n_{1},n_{2}}\left(L_{1},L_{2},l_{p1},l_{p2},\omega \right)}{k_{B}T}\right)dx_{2}}\;. \end{array}$$

The force on the restricting wall in the *x* direction is obtained by calculating the integrations and that leads to

(100) $$\begin{array}{c} \langle f_{x}^{PW,n_{1},n_{2}}\rangle =k_{B}T\frac{\sum_{n_{1}=0}^{n_{1}=1}\sum_{n_{2}=0}^{n_{2}=1}d_{x,1}^{PW}\left(n_{1},n_{2}\right)}{\sum_{n_{1}=0}^{n_{1}=1}\sum_{n_{2}=0}^{n_{2}=1}d_{x,2}^{PW}\left(n_{1},n_{2}\right)}\;, \end{array}$$

where

(101) $$\begin{array}{c} d_{x,1}^{PW}\left(n_{1},n_{2}\right)=e^{-\frac{{\left(\left(L_{1}+L_{2}\right)\cos\left(\omega \right)-\delta_{x}\right)}^{2}}{{\hat{\sigma^{x}}}_{n_{1},n_{2}}}}e^{-n_{1}\;{\tilde{\epsilon }}_{1}-n_{2}\;{\tilde{\epsilon }}_{2}} \end{array}$$

and

(102) $$\begin{array}{c} d_{x,2}^{PW}(n_{1},n_{2})=\\ \;\;\;\frac{1}{2}\sqrt{\pi {\hat{\sigma^{x}}}_{n_{1},n_{2}}}\left(1-\mathrm{erf}\left(\frac{\left(L_{1}+L_{2}\right)\cos\left(\omega \right)-\delta_{x}}{\sqrt{{\hat{\sigma^{x}}}_{n_{1},n_{2}}}}\right)\right)e^{-n_{1}\;{\tilde{\epsilon }}_{1}-n_{2}\;{\tilde{\epsilon }}_{2}}\;. \end{array}$$

The average of the state occupation numbers in the presence of the restricting wall in the *x* direction can be calculated by the following integration:

(103) $$\begin{array}{c} {\langle n_{i}\rangle }_{x}^{PW}=\frac{\sum_{n_{1}=0}^{n_{1}=1}\sum_{n_{2}=0}^{n_{2}=1}\int_{-\infty }^{\delta_{x}}\left(n_{i}\right)\exp\left(\frac{-H_{x}^{n_{1},n_{2}}\left(L_{1},L_{2},l_{p1},l_{p2},\omega \right)}{k_{B}T}\right)dx_{2}}{\sum_{n_{1}=0}^{n_{1}=1}\sum_{n_{2}=0}^{n_{2}=1}\int_{-\infty }^{\delta_{x}}\exp\left(\frac{-H_{x}^{n_{1},n_{2}}\left(L_{1},L_{2},l_{p1},l_{p2},\omega \right)}{k_{B}T}\right)dx_{2}} \end{array}$$

The result of the integration is

(104) $$\begin{array}{c} {\langle n_{i}\rangle }_{x}^{PW}=\frac{\sum_{n_{1}=0}^{n_{1}=1}\sum_{n_{2}=0}^{n_{2}=1}\left(n_{i}\right)\sqrt{{\hat{\sigma^{x}}}_{n_{1},n_{2}}}\left(C_{n_{1},n_{2}}^{x_{2}}-1\right)e^{-n_{1}\;{\tilde{\epsilon }}_{1}-n_{2}\;{\tilde{\epsilon }}_{2}}}{\sum_{n_{1}=0}^{n_{1}=1}\sum_{n_{2}=0}^{n_{2}=1}\sqrt{{\hat{\sigma^{x}}}_{n_{1},n_{2}}}\left(C_{n_{1},n_{2}}^{x_{2}}-1\right)e^{-n_{1}\;{\tilde{\epsilon }}_{1}-n_{2}\;{\tilde{\epsilon }}_{2}}}\;, \end{array}$$

where

(105) $$\begin{array}{c} C_{n_{1},n_{2}}^{x_{2}}=\mathrm{erf}\left(\frac{\left(L_{1}+L_{2}\right)\cos\left(\omega \right)-\delta_{x}}{\sqrt{{\hat{\sigma^{x}}}_{n_{1},n_{2}}}}\right) \end{array}$$

**Figure 6:**
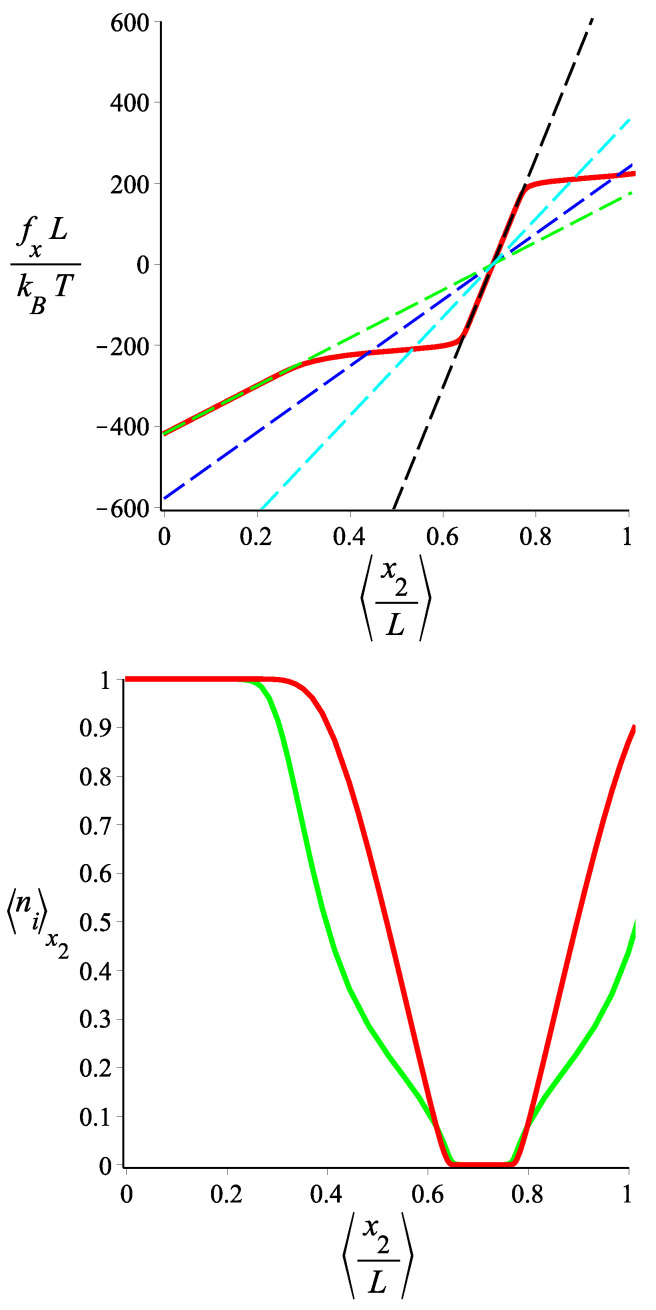
**Upper** panel: The force-extension relation of a grafted filament with two blocks in the fixed force ensemble in the *x* direction is shown. The red curve is associated with the filament with fluctuating persistence lengths. The persistence lengths of the first block and the second one fluctuate between the following values: $\left\{l_{ps1}=3000,l_{ps2}=1000\right\}$ and $\left\{l_{ps1}^{\prime }=2500,l_{ps2}^{\prime }=300\right\}$, respectively, and the curve (red curve) is given by Equation (92). The black, blue, cyan, and green dashed lines are associated with the filaments with two blocks with fixed persistence lengths $\left\{l_{p1}=l_{ps1},l_{p2}=l_{ps1}^{\prime }\right\}$, $\left\{l_{p1}=l_{ps1},l_{p2}=l_{ps2}^{\prime }\right\}$, $\left\{l_{p1}=l_{ps2},l_{p2}=l_{ps1}^{\prime }\right\}$, and $\left\{l_{p1}=l_{ps2},l_{p2}=l_{ps2}^{\prime }\right\}$, respectively, which are given by Equation (72). **Lower** panel: The average of the state occupation numbers, $n_{1},n_{2}$ (the green and the red curves are associated with ${\langle n_{1}\rangle }_{x_{2}}$ and ${\langle n_{2}\rangle }_{x_{2}}$, respectively) are shown as a function of the average position of the tip of the filament in the *x* direction. They are calculated by combining Equation (97) and (92). The fixed parameters are ω=π/4, $\tilde{\epsilon_{1}}=12$, $\tilde{\epsilon_{2}}=20$ for both panels. The contour lengths of the blocks of the filaments in the curves in both panels are fixed: $L_{1}=1$, $L_{2}=2$ and $L\equiv L_{1}+L_{2}$.

**Figure 7:**
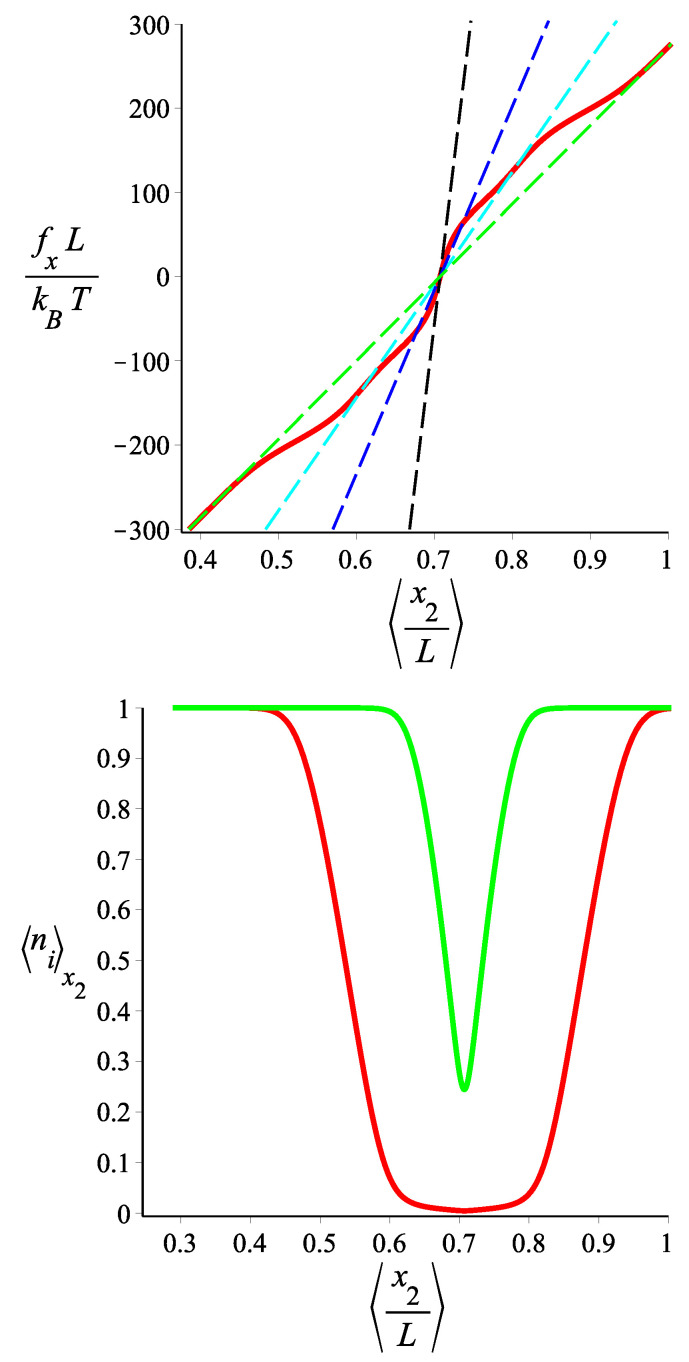
**Upper** panel: The force-extension relation of the tip of the filament with two blocks in the fixed force ensemble in the *x* coordinate is shown. The red curve is associated with the filament with fluctuating (two-state) persistence lengths. The persistence lengths of the first block and of the second one fluctuate between the following values: $\left\{l_{ps1}=800,l_{ps2}=1000\right\}$ and $\left\{l_{ps1}^{\prime }=7000,l_{ps2}^{\prime }=800\right\}$, respectively, and the curve (the red curve) is given by Equation (92). The black, blue, cyan, and green dashed lines are associated with the filaments with two blocks having fixed persistence lengths, $\left\{l_{p1}=l_{ps1},l_{p2}=l_{ps1}^{\prime }\right\}$, $\left\{l_{p1}=l_{ps1},l_{p2}=l_{ps2}^{\prime }\right\}$, $\left\{l_{p1}=l_{ps2},l_{p2}=l_{ps1}^{\prime }\right\}$, and $\left\{l_{p1}=l_{ps2},l_{p2}=l_{ps2}^{\prime }\right\}$, respectively, which are given by Equation (72). **Lower** panel: The average of the state occupation numbers, $n_{1},n_{2}$ (the green and the red curves are associated with ${\langle n_{1}\rangle }_{x_{2}}$ and ${\langle n_{2}\rangle }_{x_{2}}$, respectively) are shown as a function of the average position of the tip of the filament in the *x* direction. They are calculated from the combination of Equation (97) and Equation (92). The fixed parameters are ω=π/4, $\tilde{\epsilon_{1}}=2$, $\tilde{\epsilon_{2}}=6$ for both panels. The contour lengths of the blocks of the filament in the curves in both panels are fixed: $L_{1}=1$, $L_{2}=2$, and $L\equiv L_{1}+L_{2}$.

**Figure 8:**
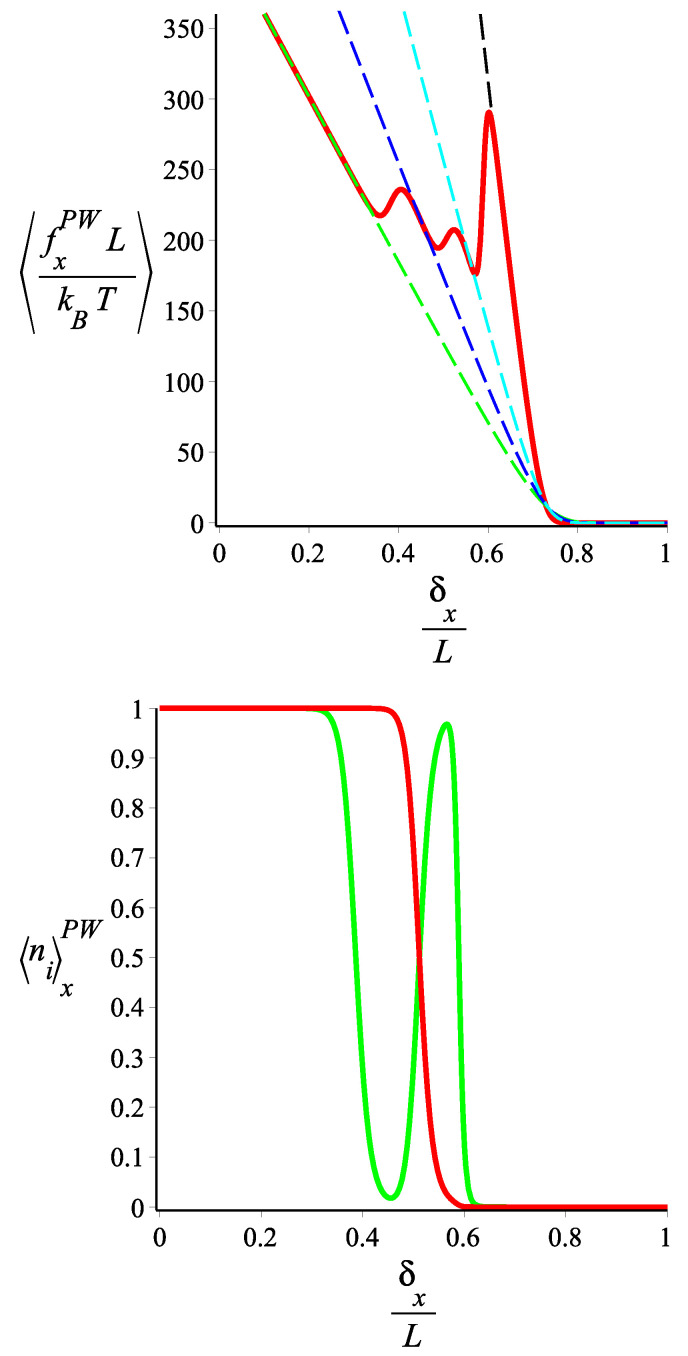
**Upper** panel: the average of the force exerted by the tip of a filament with two blocks to the confining wall in the *x* coordinate is shown. The red curve is associated with the filament with fluctuating persistence lengths. The persistence lengths of the first block and of the second one fluctuate between the following values: $\left\{l_{ps1}=3000,l_{ps2}=1000\right\}$ and $\left\{l_{ps1}^{\prime }=2500,l_{ps2}^{\prime }=300\right\}$, respectively, and the curve (the red curve) is given by Equation (100). The black, blue, cyan, and green dashed lines are associated with the filament having two blocks with fixed persistence lengths, $\left\{l_{p1}=l_{ps1},l_{p2}=l_{ps1}^{\prime }\right\}$, $\left\{l_{p1}=l_{ps1},l_{p2}=l_{ps2}^{\prime }\right\}$, $\left\{l_{p1}=l_{ps2},l_{p2}=l_{ps1}^{\prime }\right\}$, and $\left\{l_{p1}=l_{ps2},l_{p2}=l_{ps2}^{\prime }\right\}$, respectively, which are given by Equation (75). **Lower** panel: The average of the state occupation numbers, $n_{1},n_{2}$ (the green and the red curves are associated with ${\langle n_{1}\rangle }_{x_{2}}^{PW}$, and ${\langle n_{2}\rangle }_{x_{2}}^{PW}$ respectively) are shown as a function of the position of the wall in the *x* coordinate. They are calculated from Equation (104). The fixed parameters are ω=π/4, $\tilde{\epsilon_{1}}=12$, $\tilde{\epsilon_{2}}=20$ for both panels. The contour lengths of the blocks of the filaments in all curves of the both panels are fixed: $L_{1}=1$, $L_{2}=2$ and $L\equiv L_{1}+L_{2}$.

**Figure 9:**
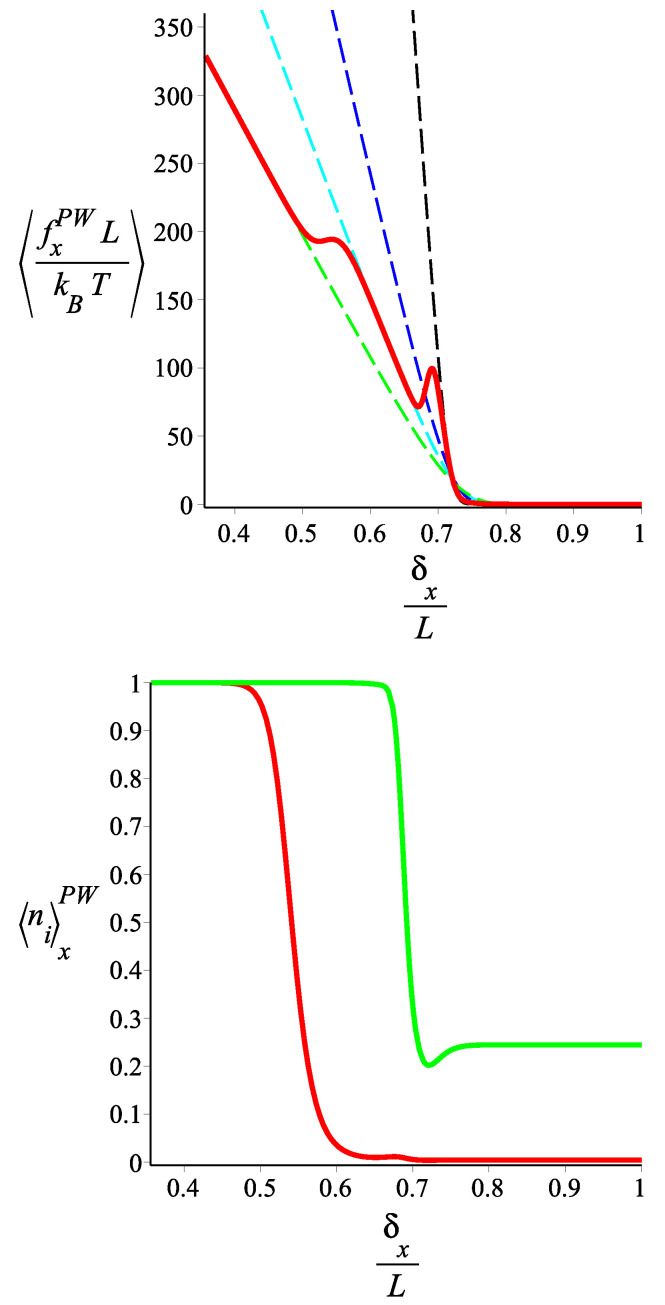
**Upper** panel: the average of the force exerted by the tip of the filament with two blocks on the restricting wall in the *x* coordinate. The red curve is associated with the filament with fluctuating persistence lengths. The persistence lengths of first block and the second one fluctuate between the following values: $\left\{l_{ps1}=800,l_{ps2}=1000\right\}$ and $\left\{l_{ps1}^{\prime }=7000,l_{ps2}^{\prime }=800\right\}$, respectively, and the curve (the red curve) is given by Equation (100). The black, blue, cyan, and green dashed lines are associated with the filaments with two blocks having fixed persistence lengths, $\left\{l_{p1}=l_{ps1},l_{p2}=l_{ps1}^{\prime }\right\}$, $\left\{l_{p1}=l_{ps1},l_{p2}=l_{ps2}^{\prime }\right\}$, $\left\{l_{p1}=l_{ps2},l_{p2}=l_{ps1}^{\prime }\right\}$, and $\left\{l_{p1}=l_{ps2},l_{p2}=l_{ps2}^{\prime }\right\}$, respectively, which are given by Equation (75). **Lower** panel: The average of the state numbers, $n_{1},n_{2}$ (the green and the red curves are associated with ${\langle n_{1}\rangle }_{x_{2}}^{PW}$, and ${\langle n_{2}\rangle }_{x_{2}}^{PW}$, respectively) are shown as a function of the position of the wall in the *x* coordinate. They are calculated from Equation (104). The fixed parameters are ω=π/4, $\tilde{\epsilon_{1}}=2$, $\tilde{\epsilon_{2}}=6$ for both panels. The contour lengths of the blocks of the filaments in all curves of both panels are fixed: $L_{1}=1$, $L_{2}=2$, and $L\equiv L_{1}+L_{2}$.

In Figure 6 and Figure 7, we show the force-extension relation, in the Gibbs ensemble, of the two-block fluctuating-persistence-length grafted filament, for two different sets of parameters. We notice that the elastic response (stiffness) of the filament changes significantly with the applied force. For some values of the force, the response corresponds to a block copolymer with a well-defined bending stiffness in each block, whereas for some other values, the response is a statistical mixture of states. Because we are in the Gibbs ensemble (see Appendix A of Ref [29]), the force-extension curve, despite its irregularity, is monotonic and the extensivity and compressibility are positive. It is interesting to note, in Figure 6, that around certain values of the force (which is the control parameter), the differential stiffness of the filament (given by the slope of the force-extension curve) becomes much smaller than that of any well-defined state of the copolymer. By “well-defined state” we mean a filament with two blocks at fixed values of the bending stiffness. In Figure 8 and Figure 9, we show how the average force that the tip of the grafted filament exerts on a confining wall changes with the position of the wall. This is applied to two sets of parameters. Notice that the filament parameters of Figure 8 and Figure 9 are the same as those of Figure 6 and Figure 7, respectively. Because the wall position is fixed, the calculation is performed in the Helmholtz ensemble. As we change the wall position, the filament jumps from one well-defined bending state to another well-defined bending state in a “spiky” fashion, with regions of negative compressibility, similar to the elastic response of other two-state systems in the Helmholtz ensemble. We point out that, as we see in Figure 9, certain well-defined states may be skipped in the force-compression curve, depending on the choice of parameters.

We now present the analysis of the grafted filament’s response to a force (or deformation) in the *y* direction. The Hamiltonian in Equation (87) gives the Helmholtz free energy of the tip of the filament in the *y* direction (for a given choice of states),

(106) $$\begin{array}{cc} H_{y}^{n_{1},n_{2}} & (L_{1},L_{2},l_{ps1}+n_{1}\left(l_{ps2}-l_{ps1}\right),l_{ps1}^{\prime }+n_{2}\left(l_{ps2}^{\prime }-l_{ps1}^{\prime }\right),\omega )=\\ & +\frac{k_{B}T{\left(y_{2}-\left(L_{1}+L_{2}\right)\sin\left(\omega \right)\right)}^{2}}{\sigma_{y}\left(L_{1},L_{2},l_{ps1}+n_{1}\left(l_{ps2}-l_{ps1}\right),l_{ps1}^{\prime }+n_{2}\left(l_{ps2}^{\prime }-l_{ps1}^{\prime }\right),\omega \right)}\\ & +n_{1}\epsilon_{1}+n_{2}\epsilon_{2}\;, \end{array}$$

which leads to an effective Hamiltonian in the presence of a fixed external force in the *y* direction:

(107) $$\begin{array}{cc} H_{y}^{\prime n_{1},n_{2}} & (L_{1},L_{2},l_{ps1}+n_{1}\left(l_{ps2}-l_{ps1}\right),l_{ps1}^{\prime }+n_{2}\left(l_{ps2}^{\prime }-l_{ps1}^{\prime }\right),\omega )=\\ & +\frac{k_{B}T{\left(y_{2}-\left(L_{1}+L_{2}\right)\sin\left(\omega \right)\right)}^{2}}{\sigma_{y}\left(L_{1},L_{2},l_{ps1}+n_{1}\left(l_{ps2}-l_{ps1}\right),l_{ps1}^{\prime }+n_{2}\left(l_{ps2}^{\prime }-l_{ps1}^{\prime }\right),\omega \right)}\\ & -f_{y}\left(y_{2}-\left(L_{1}+L_{2}\right)\sin\left(\omega \right)\right)+n_{1}\epsilon_{1}+n_{2}\epsilon_{2}\;. \end{array}$$

The force-extension relation in the *y* direction in the fixed force ensemble is obtained by calculating the following Boltzmann average,

(108) $$\begin{array}{c} \langle y_{2}\rangle =\frac{\sum_{n_{1}=0}^{n_{1}=1}\sum_{n_{2}=0}^{n_{2}=1}\int_{-\infty }^{+\infty }\left(y_{2}\right)\exp\left(-\frac{H_{y}^{\prime n_{1},n_{2}}}{k_{B}T}\right)dy_{2}}{\sum_{n_{1}=0}^{n_{1}=1}\sum_{n_{2}=0}^{n_{2}=1}\int_{-\infty }^{+\infty }\exp\left(-\frac{H_{y}^{\prime n_{1},n_{2}}}{k_{B}T}\right)dy_{2}}\;, \end{array}$$

where we introduce the following expression as an abbreviation:

(109) $$H_{y}^{\prime n_{1},n_{2}}\equiv H_{y}^{\prime n_{1},n_{2}}\left(L_{1},L_{2},l_{ps1}+n_{1}\left(l_{ps2}-l_{ps1}\right),l_{ps1}^{\prime }+n_{2}\left(l_{ps2}^{\prime }-l_{ps1}^{\prime }\right),\omega \right)\;.$$

The ensuing force-extension relation in the *y* coordinate (in fixed force ensemble) is

(110) $$\langle y_{2}\rangle -\left(L_{1}+L_{2}\right)\sin\left(\omega \right)=\frac{d_{y,1}}{d_{y,2}}\;,$$

where

(111) $$\begin{array}{c} d_{y,1}=\frac{\sqrt{\pi }\tilde{f_{y}}}{2}\sum_{n_{1}=0}^{n_{1}=1}\sum_{n_{2}=0}^{n_{2}=1}{\left({\hat{\sigma^{y}}}_{n_{1},n_{2}}\right)}^{3/2}e^{\left(-n_{1}\;{\tilde{\epsilon }}_{1}-n_{2}\;{\tilde{\epsilon }}_{2}+1/4\;{\tilde{f_{y}}}^{2}{\hat{\sigma^{y}}}_{n_{1},n_{2}}\right)} \end{array}$$

and

(112) $$\begin{array}{c} d_{y,2}=\sum_{n_{1}=0}^{n_{1}=1}\sum_{n_{2}=0}^{n_{2}=1}{\left(\pi {\hat{\sigma^{y}}}_{n_{1},n_{2}}\right)}^{\frac{1}{2}}e^{-n_{1}\;{\tilde{\epsilon }}_{1}-n_{2}\;{\tilde{\epsilon }}_{2}+1/4\;{\tilde{f_{y}}}^{2}{\hat{\sigma^{y}}}_{n_{1},n_{2}}} \end{array}$$

and ${\hat{\sigma^{y}}}_{n_{1},n_{2}}$ is a 2×2 matrix with the following elements:

(113) $${\hat{\sigma^{y}}}_{0,0}=\sigma_{y}\left(L_{1},L_{2},l_{ps1},l_{ps1}^{\prime },\omega \right)$$

$${\hat{\sigma^{y}}}_{0,1}=\sigma_{y}\left(L_{1},L_{2},l_{ps1},l_{ps2}^{\prime },\omega \right)$$

$${\hat{\sigma^{y}}}_{1,0}=\sigma_{y}\left(L_{1},L_{2},l_{ps2},l_{ps1}^{\prime },\omega \right)$$

$${\hat{\sigma^{y}}}_{1,1}=\sigma_{y}\left(L_{1},L_{2},l_{ps2},l_{ps2}^{\prime },\omega \right)$$

Also, the average of the state occupation numbers $n_{1}$ and $n_{2}$ in the fixed force ensemble in the *y* coordinate can be calculated by the following expression:

(114) $$\begin{array}{c} {\langle n_{i}\rangle }_{y_{2}}=\frac{\sum_{n_{1}=0}^{n_{1}=1}\sum_{n_{2}=0}^{n_{2}=1}\int_{-\infty }^{+\infty }\left(n_{i}\right)\exp\left(-\frac{H_{y}^{\prime n_{1},n_{2}}}{k_{B}T}\right)dy_{2}}{\sum_{n_{1}=0}^{n_{1}=1}\sum_{n_{2}=0}^{n_{2}=1}\int_{-\infty }^{+\infty }\exp\left(-\frac{H_{y}^{\prime n_{1},n_{2}}}{k_{B}T}\right)dy_{2}}\;. \end{array}$$

The integration leads to

(115) $$\begin{array}{c} {\langle n_{i}\rangle }_{y_{2}}=\frac{\sum_{n_{1}=0}^{n_{1}=1}\sum_{n_{2}=0}^{n_{2}=1}\left(\left(n_{i}\right)e^{-n_{1}\;{\tilde{\epsilon }}_{1}-n_{2}\;{\tilde{\epsilon }}_{2}+1/4\;{\tilde{f_{y}}}^{2}{\hat{\sigma^{y}}}_{n_{1},n_{2}}}\sqrt{{\hat{\sigma^{y}}}_{n_{1},n_{2}}}\right)}{\sum_{n_{1}=0}^{n_{1}=1}\sum_{n_{2}=0}^{n_{2}=1}\left(e^{-n_{1}\;{\tilde{\epsilon }}_{1}-n_{2}\;{\tilde{\epsilon }}_{2}+1/4\;{\tilde{f_{y}}}^{2}{\hat{\sigma^{y}}}_{n_{1},n_{2}}}\sqrt{{\hat{\sigma^{y}}}_{n_{1},n_{2}}}\right)}\;. \end{array}$$

The force exerted by the tip of the filament on a confining wall in the *y* direction is given by taking the derivative of the corresponding free energy,

(116) $$\begin{array}{c} \langle f_{y}^{PW,n_{1},n_{2}}\rangle =k_{B}T\frac{d}{d\delta_{y}}\ln\left(Z_{y}^{n_{1},n_{2}}\left(\delta_{y}\right)\right) \end{array}$$

where $\delta_{y}$ is the distance of the wall from the grafting point in the *y* direction and

(117) $$\begin{array}{c} Z_{y}^{n_{1},n_{2}}\left(\delta_{y}\right)=\frac{\sum_{n_{1}=0}^{n_{1}=1}\sum_{n_{2}=0}^{n_{2}=1}\int_{-\infty }^{\delta_{y}}\exp\left(\frac{-H_{y}^{n_{1},n_{2}}\left(L_{1},L_{2},l_{p1},l_{p2},\omega \right)}{k_{B}T}\right)dy_{2}}{\sum_{n_{1}=0}^{n_{1}=1}\sum_{n_{2}=0}^{n_{2}=1}\int_{-\infty }^{+\infty }\exp\left(\frac{-H_{y}^{n_{1},n_{2}}\left(L_{1},L_{2},l_{p1},l_{p2},\omega \right)}{k_{B}T}\right)dy_{2}}\;. \end{array}$$

The result of the calculation for the average force on the restricting wall in the *y* direction is

(118) $$\begin{array}{c} \langle f_{y}^{PW,n_{1},n_{2}}\rangle =k_{B}T\frac{\sum_{n_{1}=0}^{n_{1}=1}\sum_{n_{2}=0}^{n_{2}=1}d_{y,1}^{PW}\left(n_{1},n_{2}\right)}{\sum_{n_{1}=0}^{n_{1}=1}\sum_{n_{2}=0}^{n_{2}=1}d_{y,2}^{PW}\left(n_{1},n_{2}\right)}\;, \end{array}$$

where

(119) $$\begin{array}{c} d_{y,1}^{PW}\left(n_{1},n_{2}\right)=e^{-\frac{{\left(\left(L_{1}+L_{2}\right)\sin\left(\omega \right)-\delta_{y}\right)}^{2}}{{\hat{\sigma^{y}}}_{n_{1},n_{2}}}}e^{-n_{1}\;{\tilde{\epsilon }}_{1}-n_{2}\;{\tilde{\epsilon }}_{2}} \end{array}$$

and

(120) $$\begin{array}{c} d_{y,2}^{PW}(n_{1},n_{2})=\\ \;\;\;\;\;\frac{1}{2}\sqrt{\pi \hat{\sigma^{y}}n_{1},n_{2}}\left(1-\mathrm{erf}\left(\frac{\left(L_{1}+L_{2}\right)\sin\left(\omega \right)-\delta_{y}}{\sqrt{{\hat{\sigma^{y}}}_{n_{1},n_{2}}}}\right)\right)e^{-n_{1}\;{\tilde{\epsilon }}_{1}-n_{2}\;{\tilde{\epsilon }}_{2}} \end{array}$$

The average of the state occupation numbers in the presence of the restricting wall in the *y* direction can be calculated by the following integration:

(121) $$\begin{array}{c} {\langle n_{i}\rangle }_{y}^{PW}=\frac{\sum_{n_{1}=0}^{n_{1}=1}\sum_{n_{2}=0}^{n_{2}=1}\int_{-\infty }^{\delta_{y}}\left(n_{i}\right)\exp\left(\frac{-H_{y}^{n_{1},n_{2}}\left(L_{1},L_{2},l_{p1},l_{p2},\omega \right)}{k_{B}T}\right)dy_{2}}{\sum_{n_{1}=0}^{n_{1}=1}\sum_{n_{2}=0}^{n_{2}=1}\int_{-\infty }^{\delta_{y}}\exp\left(\frac{-H_{y}^{n_{1},n_{2}}\left(L_{1},L_{2},l_{p1},l_{p2},\omega \right)}{k_{B}T}\right)dy_{2}} \end{array}$$

The result of the integration gives

(122) $$\begin{array}{c} {\langle n_{i}\rangle }_{y}^{PW}=\frac{\sum_{n_{1}=0}^{n_{1}=1}\sum_{n_{2}=0}^{n_{2}=1}\left(n_{i}\right)\sqrt{{\hat{\sigma^{y}}}_{n_{1},n_{2}}}\left(C_{n_{1},n_{2}}^{y_{2}}-1\right)e^{-n_{1}\;{\tilde{\epsilon }}_{1}-n_{2}\;{\tilde{\epsilon }}_{2}}}{\sum_{n_{1}=0}^{n_{1}=1}\sum_{n_{2}=0}^{n_{2}=1}\sqrt{{\hat{\sigma^{y}}}_{n_{1},n_{2}}}\left(C_{n_{1},n_{2}}^{y_{2}}-1\right)e^{-n_{1}\;{\tilde{\epsilon }}_{1}-n_{2}\;{\tilde{\epsilon }}_{2}}}\;, \end{array}$$

where

(123) $$\begin{array}{c} C_{n_{1},n_{2}}^{y_{2}}=\mathrm{erf}\left(\frac{\left(L_{1}+L_{2}\right)\sin\left(\omega \right)-\delta_{y}}{\sqrt{{\hat{\sigma^{y}}}_{n_{1},n_{2}}}}\right)\;. \end{array}$$

## 6. Discussion and Conclusions

In this article, we used analytical methods to investigate the elasticity of a grafted weakly bending (rod-like) semiflexible filament having fluctuating bending stiffness. We considered two types of filaments, namely, a homopolymer fluctuating between two values of its bending stiffness, and a copolymer consisting of two blocks, where each block can fluctuate independently between two values of its bending stiffness. We also considered two types of elastic deformation, namely, the response to a point force exerted at the tip of the filament and the response to a planar, rigid confining wall. Our analysis revealed a very rich and interesting elastic behavior.

In the Gibbs ensemble, the differential stiffness (slope of the force-extension curve) can change significantly as we vary the applied force and it can take values much smaller than those of the “pure” (non-fluctuating) states. For the two-state homopolymer, we have explicitly shown the inequivalence between the Gibbs and the Helmholtz ensemble. Whereas the slope of the force-extension curve is always positive in the Gibbs ensemble (as prescribed by the second law of the thermodynamics, see Ref. [29]), for a certain choice of parameters, the force-extension relation may have regions of negative slope (extensibility or compressibility). We have to point out a novelty of our system. In previous studies of bistable polymer systems in the Helmholtz ensemble, negative extensibility [21,22,23,24,25,26,27,28,29] or negative compressibility [16,17] have already been reported. Because of the positional-orientational coupling of the grafted rod-like bistable filament, we can observe both a negative extensibility and a negative compressibility in the same curve (Figure 3). The average force exerted by the fluctuating tip on a confining planar wall is calculated in the Helmholtz ensemble, and the resulting force-compression curve exhibits a spiky form with regions of negative differential compressibility.

There is another interesting difference between the weakly bending, rod-like rWLC analysed in this article and the strongly stretched rWLC analysed in Ref. [29]. In the strongly stretched rWLC, strong stretching leads to stress/strain stiffening of the filament (the state with larger bending stiffness becomes favored). On the other hand, in the model studied here, strong force/strain leads to softening of the filament (the state with smaller bending stiffness becomes dominant). We see that both in the force-extension relation for a point force at the tip and in the force-compression relation for the response to the compressing wall. The apparent contradiction can be explained, if we realize that the high bending stiffness state favors straight configurations in both cases. In the case of strong stretching, a stronger force favors configurations compatible with high bending stiffness, whereas in the present case, a higher force causes bending which is favored by a lower bending stiffness.

Our analytical calculations are based on the weakly bending approximation which yields Gaussian probabilities and linear force-extension relations (the only nonlinearity stems from the two-state blocks). It is known that the local inextensibility constraint of the WLC causes significant nonlinear response under strong stretching. As the WLC tends to full extension, the corresponding tensile force diverges. The onset of this nonlinearity in the WLC occurs when $\sqrt{\kappa /f}\leq L$ [39,40]. In Figure 3 and Figure 4, we have chosen the parameters so that the interesting crossover from the stiffer to the softer state can happen within the linear regime of the WLC. More specifically, the onset of a nonlinear response due to the local inextensibility of the WLC occurs at a value of the force $f_{x}\approx l_{ps2}k_{B}T/\left(2L^{2}\cos\omega \right)\approx 339$ in our units ($k_{B}T=1$, L=1). The curves in other figures, at some values, go beyond the linear (Gaussian) approximation. However, our theoretical analysis clearly shows that the mechanism behind the behavior presented here is robust, and a more realistic model for the elasticity of the single-state WLC homopolymer would only make a quantitative difference.

As we mentioned in the Introduction, a possible physical realization of our model of a homopolymer with fluctuating bending stiffness would be a weakly bending DNA or carbon nanotube which gets reversibly hybridized by a DNA strand along its contour (the hybridized state will have higher bending stiffness). We would like to point out the relevance of our analysis to the metamaterial behavior of the Huxley–Simmons model of muscle elasticity, proposed by Caruel et al. [41,42,43,44,45]. The value of the energy parameter that we use in Figure 3 and Figure 4, $\tilde{\epsilon }=10$ agrees with that of 50±10zJ for the snap-spring muscle model of Caruel and Truskinovsky [46]. Those studies are based on a very coarse-grained model of the muscle as consisting of bistable spring units. As the sarcomere consists of actomyosin bundles which themselves can be viewed as a semiflexible filaments of fluctuating bending stiffness, our analysis may provide a path towards a more microscopic theoretical understanding.

In this article, we also analysed the case of a filament with two two-state blocks. A possible physical realization would be that of a DNA or carbon nanotubes hybridized on different parts by different DNA strands. The predicted elastic behavior is very rich, and we hope that our theoretical study may trigger further experimental or engineering investigations on such systems.

## Data Availability

Not applicable.
